# Snow alga *Sanguina aurantia* as revealed through de novo genome assembly and annotation

**DOI:** 10.1093/g3journal/jkae181

**Published:** 2024-08-02

**Authors:** Breanna B Raymond, Pierre Guenzi-Tiberi, Eric Maréchal, Lynne M Quarmby

**Affiliations:** Department of Molecular Biology and Biochemistry, Simon Fraser University, 8888 University Drive, Burnaby, BCBC V5A 1S6, Canada; Laboratoire de Physiologie Cellulaire et Végétale, CNRS, CEA, INRAE, Université Grenoble Alpes, IRIG, CEA Grenoble, 17 Avenue des Martyrs, 38000 Grenoble, France; Laboratoire de Physiologie Cellulaire et Végétale, CNRS, CEA, INRAE, Université Grenoble Alpes, IRIG, CEA Grenoble, 17 Avenue des Martyrs, 38000 Grenoble, France; Department of Molecular Biology and Biochemistry, Simon Fraser University, 8888 University Drive, Burnaby, BCBC V5A 1S6, Canada

**Keywords:** genome assembly, snow algae, Chlorophyta, culture, psychrophilic, RNA-Seq, Hi-C, *Sanguina aurantia*, variants, nanopore

## Abstract

To thrive on melting alpine and polar snow, some Chlorophytes produce an abundance of astaxanthin, causing red blooms, often dominated by genus *Sanguina*. The red cells have not been cultured, but we recently grew a green biciliate conspecific with *Sanguina aurantia* from a sample of watermelon snow. This culture provided source material for Oxford Nanopore Technology and Illumina sequencing. Our assembly pipeline exemplifies the value of a hybrid long- and short-read approach for the complexities of working with a culture grown from a field sample. Using bioinformatic tools, we separated assembled contigs into 2 genomic pools based on a difference in GC content (57.5 and 55.1%). We present the data as 2 assemblies of *S. aurantia* variants but explore other possibilities. High-throughput chromatin conformation capture analysis (Hi-C sequencing) was used to scaffold the assemblies into a 96-Mb genome designated as “A” and a 102-Mb genome designated as “B.” Both assemblies are highly contiguous: genome A consists of 38 scaffolds with an N50 of 5.4 Mb, while genome B has 50 scaffolds with an N50 of 6.4 Mb. RNA sequencing was used to improve gene annotation.

## Introduction

During the melt season, snow algae form expansive red blooms known as watermelon snow on alpine and polar snowfields (Hoham and Remias 2020). The snow algae environment is characterized by temperatures from below freezing to around 0°C during the day when light intensity can be up to 5,000 µmol m^−2^ s^−1^ photosynthetically active irradiation. An important challenge for algae in a high-light and low-temperature environment is the ability to photosynthesize and maintain photostasis (Cvetkovska *et al*. 2017). Other challenges include UV radiation when close to the snowpack surface, risk of desiccation, and means to colonize each year's new snow. The lack of a high-quality snow algal genome has limited our understanding of how snow algae thrive despite these and other challenges of life on seasonal snow.

The Chlorophytes comprising red blooms synthesize astaxanthin. The red cells darken the snow surface, decrease albedo, and accelerate snowmelt (Engstrom *et al*. 2022; Engstrom and Quarmby 2023). A dominant genus in watermelon snow is *Sanguina* (Brown and Tucker 2020; Engstrom *et al*. 2020; Procházková *et al*. 2019). Historically, red spherical cells were broadly depicted as *Chlamydomonas nivalis* until the genus was deemed polyphyletic based on improved molecular techniques (Engstrom *et al*. 2024; Matsuzaki *et al*. 2015; Procházková *et al*. 2019). We now know that many cell types resembling classical morphological descriptions of *C. nivalis* are genetically distinct and require taxonomic reassessment, evidenced by the 2 novel genera *Rosetta* and *Sanguina* (Engstrom *et al*. 2024; Procházková *et al*. 2019). *Sanguina* currently consists of 2 named species *Sanguina nivaloides* and *Sanguina aurantia*, colored blood red and orange, respectively, as a consequence of their astaxanthin-to-chlorophyll-a pigment ratios (Procházková *et al*. 2019, 2021).

Until recently, no snow algal culture has genetically matched the bright red or orange cells that often dominate field samples. This has made obtaining a genome assembly for these red cells difficult. In an attempt at de novo assembly of the genome of *S. nivaloides*, Tucker and Brown (2023) applied a pooling approach to sequences derived from cells picked from field samples. Unfortunately, the quality of the data was insufficient for assembly. Furthermore, the genes they report cannot be definitively assigned to *S. nivaloides* because the cells were identified by morphology alone and diverse species of snow algae are morphologically similar (see, for example, Engstrom *et al*. 2024). After growing dozens of cultures, we eventually cultivated a green biciliate conspecific with the spherical orange, immotile cells of *S. aurantia* (Raymond *et al*. 2022). Although no transition from green to orange has been reported in laboratory conditions, the green biciliate morphology of *S. aurantia* is proposed to colonize the snow surface after over-wintering, with cells losing their cilia and expressing astaxanthin in response to conditions on the snow surface (Raymond *et al*. 2022). This transition leads to additional cell architecture changes, unraveled by 3D electron microscopy imaging (Ezzedine *et al*. 2023). These include a wrinkled plasma membrane adapted to nutrient's absorption in an oligotrophic environment, a spatial organization of thylakoids allowing the capture of light scattered in all directions, a short-term storage of carbon as starch in the chloroplast, and a long-term storage in triacylglycerol, in cytosolic lipid droplets, allowing survival during winter months (Ezzedine *et al*. 2023). Cell architecture also highlights the ongoing and sequential loading of cytosolic lipid droplets with astaxanthin, when the droplets were bound to the endoplasmic reticulum (ER), whereas this loading was complete in mature droplets undocked from the ER, or other organelles such as mitochondria (Ezzedine *et al*. 2023).

Using both Illumina and Oxford Nanopore Technology (ONT), we sequenced the culture of green *S. aurantia* cells and generated a de novo genome assembly. We identified 2 pools of genomic contigs separated based on GC content. We propose that each of these pools represents a highly contiguous and complete genome of *S. aurantia*, possibly reflecting the growth of 2 distinct cells from the original field sample (to date, we have been unable to grow cultures of this species from individual cells). We used high-throughput chromatin conformation capture (Hi-C) to refine high-quality assemblies and used RNA sequencing to verify ab initio and homology-based gene predictions. We present a reproducible workflow that highlights the complexities of de novo genome assemblies for green algae cultured directly from a field sample.

## Materials and methods

### Culture

**Fig. 1.**
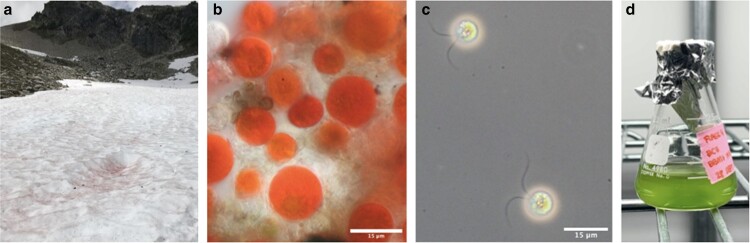
Field sample and culture images of *S. aurantia*. a) Field image of red bloom on Brandywine Mountain in British Columbia (bdw1925: 50.106285, −123.203154) where the field sample used for culture inoculation was taken. b) Microscopy image of cells in the red bloom field sample bdw1925, used for culture inoculation. c) Differential interference contrast (DIC) microscopy image of the cultured *S. aurantia* biciliate cells used for genomic sequencing. d) Flask of green biciliate *S. aurantia* cells, representative of culture used for genomic sequencing. All scale bars are 15 μm.

The in-house culture of *S. aurantia* used for sequencing was first described in Raymond *et al*. (2022) as thin-walled, green, biciliate cells designated conspecific with field samples of thick-walled orange cells based on ITS2 and *rbcL* sequences. The culture originated from a sample of red snow collected on 2019 July 24 from an alpine bloom on Brandywine Meadows in Squamish-Lillooet, British Columbia. Of note, we used this procedure to grow cultures from over 100 field samples from across British Columbia over 3 bloom seasons. Described here is the only culture we identified as genus *Sanguina*. As described in Raymond *et al*. (2022), this culture grew from a red snow sample that was collected in a sterile 50-mL centrifuge tube and transported to the laboratory packed in snow. On immediate return from the field, aliquots of the partially thawed sample were used to inoculate a 24-well plate; each well contained 0.5 mL of media and ∼0.5 mL of sample. The medium used was 4°C liquid 3N-Bold's basal medium (3 times the amount of nitrate in BBM; http://cccryo.fraunhofer.de/sources/files/medien/BBM.pdf) with added ammonium (1 mL/L; 3N-BBM + NH4). The plate was placed on a shaker under 115 μmol m^−2^ s^−1^ SunBlaster grow lights (bulb model F24TK 24W HO 6400K) at 4°C. After 2 weeks, green growth was observed from one sample (bdw1925: 50.106285, −123.203154) and cells transferred to a 25-mL Erlenmeyer flask with 10 mL of 3N-BBM + NH_4_ (Fig. 1). Cells from the top of the liquid media are passaged to new media every month to maintain a healthy, viable culture. As the cultures are not axenic, we perform regular rounds of washing and serial dilutions to minimize growth of unattached bacteria. We have attempted to grow the culture from a single cell but have been unsuccessful growing colonies on agar plates.

Upon return to the laboratory, an aliquot of the field sample, bdw1925, was viewed under an Axioscope 2 + (Zeiss, Jena, Germany) and photographed with a Canon EOS T6 camera (Canon, Tokyo, Japan) (Fig. 1). In Raymond *et al*. (2022), we used single-cell PCR techniques and the genetic markers *rbcL* and ITS2 to sequence and identify the culture as *S. aurantia.* Using batches of 5–10 individually picked cells from the culture, all sequences of the culture returned identical for *rbcL* (*n* = 10 sequences) and ITS2 (*n* = 14 sequences) genetic markers and all cells appear uniform by light microscopy (Fig. 1; Raymond *et al*. 2022). The same approach was used to sequence single orange, immotile cells from field samples (sample don1903; MZ955646; 51.263196, −117.441067; Raymond *et al*. 2022) that were revealed to be conspecific with the culture.

### gDNA extraction, Illumina sequencing, and Oxford Nanopore sequencing

Cell lysis was performed using sonication in 100-mL falcon tubes, and in total, ∼ 4,000 mL of liquid culture was used with a concentration of ∼ 6 × 10^6^ cells/mL. Cells were harvested and DNA was isolated in succession in March 2021 using the Plant DNA Isolation Kit (magnetic bead system; Norgen Biotek Corporation) following the manual protocol, with the only modification being no bead beating during the lysis step. The amount of DNA was quantified using a Qubit fluorometer (Thermo Fisher, Massachusetts, USA), and the quality was analyzed with a NanoDrop microvolume spectrophotometer (Thermo Fisher). Multiple rounds of extraction from the culture were combined and increased in concentration using a Savant SpeedVac (Thermo Fisher) to reach a final concentration of 108 ng/mL and an A260/280 of 2.05 and A260/230 of 1.53. A second extraction was performed with the Plant DNA Isolation Kit (Norgen Biotek Corporation) using bead beating, yielding a higher DNA concentration of 154 ng/mL. Both extracted gDNA samples were submitted to Canada's Michael Smith Genome Sciences Centre (MSGSC) at BC Cancer Center for library construction and sequencing Oxford Nanopore, Illumina, and Hi-C sequencing. 100 ng of the gDNA samples was run on a pulse-field gel electrophoresis (PFGE QC) in 1% agar, 0.5 × TBE for 22 h to check DNA quality. As expected, the extraction method that omitted bead beating resulted in a less fragmented gDNA sample (Supplementary Fig. 1), and this sample, 5.7 μg in total, was used for both Oxford Nanopore and Illumina sequencing. Genomic Oxford Nanopore and PCRFree whole-genome Illumina library construction was performed at the MSGSC. The Oxford Nanopore library was run on one F9 flow cell on the PromethION (ONT, Oxford, UK). The MSGSC removed 6.0 Gb of low-quality Oxford Nanopore reads (with a *Q*-score under 7), and we received 87.2 Gb of data that passed initial quality control. Illumina paired-end (PE) 150 bp sequencing at ∼ 60 M individual reads (∼ 30 M read pairs) sequencing was performed by MSGSC on an Illumina HiSeq (Illumina, California, USA), and we obtained 18.9 Gb forward and 18.9 Gb reverse reads that passed initial quality inspection.

### Preprocessing reads

**Fig. 2.**
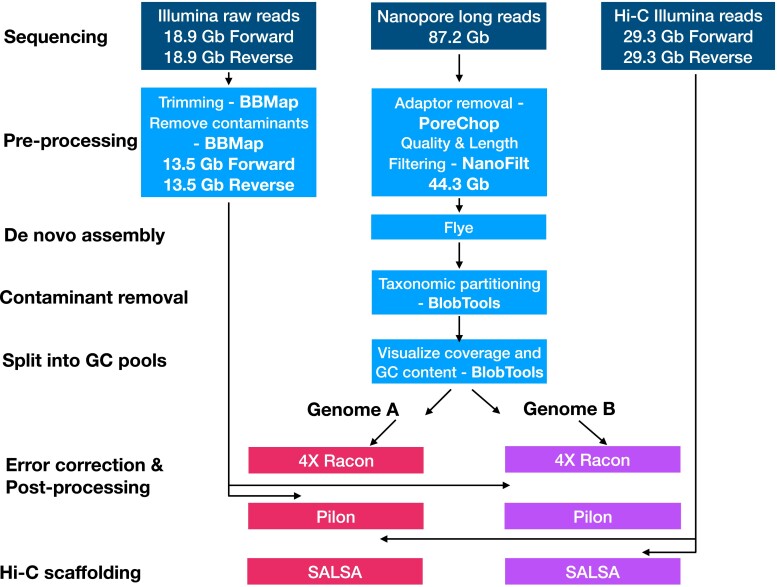
Assembly pipeline outlining the hybrid sequencing approach, processing of reads, assembly, contaminant removal, separation of Genomes A and B, polishing, and Hi-C scaffolding.

Our assembly pipeline is outlined in Fig. 2. The quality of Oxford Nanopore reads was checked between each preprocessing step using FastQC v0.12.0 (https://github.com/s-andrews/FastQC). PoreChop v0.2.4 was used to remove adaptors and chimeras with the settings “–discard_middle –threads 20” (https://github.com/rrwick/Porechop). Reads were filtered to a minimum length of 5,000 bp and a minimum *Q*-score of 10 using NanoFilt v2.8.0 “-q 10 -l 5000” (De Coster *et al*. 2018). After the filtering of Nanopore reads, we retained a total 44.3 Gb of data to use for the assembly. Nanopore read length ranged between 5,000 and 170,949 bp with an average length of 15,327.4 bp and an average quality score per sequence of 20 (FastQC; https://github.com/s-andrews/FastQC).

Illumina reads were prepared by first trimming with BBMap v38.86 (bbduk.sh; https://github.com/BioInfoTools/BBMap) using the settings “ktrim = r k = 23 mink = 11 hdist = 1 tpe tbo minlen = 50 qtrim = rl trimq = 30 maq = 33 threads = 40'. Potential contaminants were removed with BBMap (bbduk.sh; https://github.com/BioInfoTools/BBMap) using a manually curated database of all bacteria and fungi sequences on the NCBI (taken June 2021; Agarwala *et al*. 2018). After filtering, we kept 13.5 Gb forward and 13.5 Gb reverse of Illumina reads for downstream analyses. GenomeScope v1.0.0 was used to estimate the overall genome characteristics with Illumina short reads (Supplementary Fig. 2) (Vurture *et al*. 2017). An estimated genome size of 200 Mb indicates that we have an estimated 220-fold coverage of Nanopore reads and 135-fold coverage of Illumina reads. The actual read depths achieved by mapping are 135-fold for genome A and 170-fold for genome B with Nanopore data and 43-fold for genome A and 56-fold for genome B with Illumina data.

### Genome assembly

The de novo assembler Flye v2.9.1 was chosen for assembly because of its fast run time and ability to tolerate sequencing errors (Chen *et al*. 2020; Dumschott *et al*. 2020). 44.3 Gb of processed Oxford Nanopore reads was used for the Flye v2.9.1 assembly. We compared the assembly against other assemblers including MaSuRCA (Zimin *et al*. 2013), SPAdes (Bankevich *et al*. 2012), and metaFlye (Kolmogorov *et al*. 2020), but all output a more fragmented assembly.

### Assembly quality control and separating %GC content data pools

**Fig. 3.**
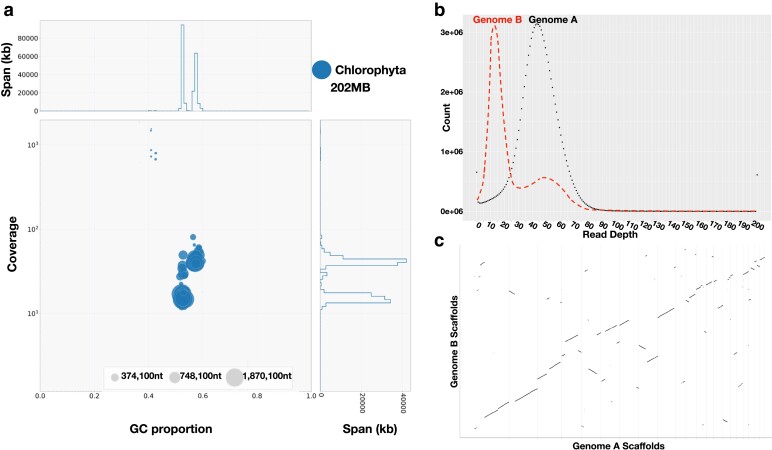
Comparison between genome A and genome B *S. aurantia* assemblies. a) Blobtools was used to visualize the quality of the assembly after contaminants were removed. A BLAST search output and alignment file of mapped Illumina reads were used as input. The 2D scatter plot is decorated with coverage and GC histograms. Contigs are represented by circles in the scatter plot, with circle diameter proportional to sequence length and colored by taxonomic affiliation. The generated blobplot displays Chlorophyta and is evidence of 2 GC pools with an associated difference in coverage. b) Once contigs were separated, a read-depth histogram of each *S. aurantia* genome was generated by mapping Illumina reads to each assembly. c) The assemblies were compared in a dot plot by aligning *S. aurantia* Genomes A and B.

We used BlobTools v1.1.1 to visualize assembly quality. To prepare 2 files for BlobTools, we first mapped Illumina reads to our assembly with BWA v0.7.17 and then searched assembled contigs against the NCBI nucleotide (nt) database using BLASTn v2.2.26 (Altschul *et al*. 1990). BlobTools generated 2 plots, a read coverage bar chart based on taxonomic assignment of contigs (Supplementary Fig. 3) and a “blobplot,” a 2D scatter plot with coverage and GC histograms colored by taxonomic affiliation (Supplementary Fig. 4). BlobTools assigned each contig to a division, and we used this for the taxonomic partitioning of our genome assembly. We identified and removed any potential bacterial contaminants, keeping only contigs whose best hit was Chlorophyta. After removing contaminant contigs, we generated a blobplot of the “clean” assembly (Fig. 3a) and used BUSCO v5.4.6 **(**Benchmarking Universal Single-Copy Orthologs) to assess the completeness of the genome with the default “–metaeuk” (Hanschen *et al*. 2020). Although our genome was largely complete with a BUSCO score of 92.8%, 65.2% of the genome content was duplicated (Supplementary Table 1, column “A”).

To interpret this unexpected feature, we mapped our Illumina reads to the genome and generated a read coverage histogram plot for each stage of the assembly using the program purge_haplotigs (Fig. 3b and Supplementary Fig. 5; https://github.com/skingan/purge_haplotigs_multiBAM; Roach *et al*. 2018). The “initial” and “clean” genome plot showed peaks at ∼ 43 × and ∼ 13 × read depths (Supplementary Fig. 5a and b), suggesting the possibility of 2 distinct genomic pools. When analyzing the GC blobplot (Fig. 3a), it is evident that there are 2 pools of GC values present in our contigs. Manual observation of GC values of contigs (Supplementary Table 2) showed there was a divide of contigs above 55% and contigs below; we chose this as our threshold for splitting the contigs into 2 genomic pools. Contigs > 55% GC content were pooled together as “A” (average assembly GC of 57.5%) and contigs < 55% GC content were pooled together as “B” (average assembly GC of 55.1%) (Supplementary Table 2).

We mapped Illumina reads onto each of the resulting 2 assemblies and found genomic pool “A” now only had one read-depth peak (43-fold) as we would expect if the assemblies represented 2 distinct genomes (Fig. 3b). Genomic pool “B” exhibits primarily 13-fold coverage, but still has a smaller second peak of 48-fold coverage, possibly due to unequal splitting of the contigs between 2 presumptive variants, recalling that the original culture was grown from an aliquot of a field sample, not from an isolated colony (Fig. 3b). We used BUSCO v5.4.6 as a quality control measure for genome completeness and found both GC pools have over 80% completeness, with a large reduction to nearly no duplication present in each genome (Supplementary Table 1). We used QUAST v5.0.2 to count the size, #contigs, and N50 of both genomic pools (Supplementary Table 1) (Gurevich *et al*. 2013). To eliminate the possibility that our culture had a second chlorophyte species present, we located the ITS2 genetic region on both assemblies and found it to be identical to *S. aurantia* (MZ955636.1). We continued the next steps with both genomic pools A and B.

### Genome polishing

GenomeQC (https://genomeqc.maizegdb.org) was used to check the quality of the draft assemblies with N50 and L50 values between each round of polishing (Manchanda *et al*. 2020). Four rounds of Racon v1.4.3 polishing were performed with the settings “-m 8 -x −6 -g −8 -w 500 -t 14” after mapping the processed Oxford Nanopore reads back to each of the draft assemblies (https://github.com/isovic/racon). The depth of Oxford Nanopore reads when polishing was 138.2 × and 127.8 × for assemblies A and B, respectively. Racon outputs a genomic consensus to correct raw reads in the initial assembly. Illumina reads were then mapped to both of the draft assemblies using BWA v0.7.17 (Li and Durbin 2010) and SAMtools v1.17 (Danecek *et al*. 2021) to prepare the format for a round of Pilon v1.23 polishing with the settings “–fix all –changes” (Walker *et al*. 2014). The depth of Illumina reads when polishing was 51.2 × and 47.3 × for assemblies A and B, respectively. Pilon searches for inconsistencies between the assembly and reads and attempts to fix single-base differences, indels, gaps, and misassembles. Although all contigs in genome B were under 55% at the time of separation (after the Flye assembly), due to the polishing methods using a consensus to correct reads, the average GC of genome B was increased from the initial assembly, 52.5%, to the final assembly, 55.1% (Supplementary Table 1), still 2.4% below the average of genome A. We separated the contigs before polishing because (i) GC bias is more prominent in Illumina sequencing when compared with Nanopore sequencing (Browne *et al*. 2020) and (ii) polishing by consensus will minimize the difference between the 2 genomes.

### Crosslinking DNA for Hi-C scaffolding

To prepare a sample for high-coverage Hi-C sequencing, a method based on high-throughput chromosome conformation capture techniques, we followed a combination of protocols described in the Arima-HiC 2.0 Mammalian Cell lines User Guide (document part number A160162 v00, June 2020; Arima Genomics Inc., California, USA) and Arima Plant Hi-C 2.0 Protocol (document part number A160163 v00, 2020 June; Arima Genomics Inc., California, USA). Arima-HiC 2.0 is an experimental workflow to capture the 3D conformation of genomes. Steps include crosslinking of chromatin to preserve structure, digestion using a restriction enzyme cocktail, labeling of the digested ends with a biotinylated nucleotide, ligation of the DNA, fragmentation of the ligated DNA, and amplification of the sequences to make a library for PE sequencing. We started with ∼2 g of pelleted algal cells in 2021 August and followed the “Nuclei Isolation” steps on page 13 of Arima Plant Hi-C 2.0 Protocol. We performed a semi-pure plant nuclei extraction using the CelLytic Plant Nuclei Extraction kit (Sigma Aldrich) according to the manufacturer’s recommendations as described in the Arima Plant Hi-C 2.0 Protocol. Next, we followed the “Crosslinking—Low-Input” protocol on page 14 of the Arima Hi-C 2.0 Mammalian Cell lines User Guide. Lastly, we followed the “Estimating Input Amount” protocol on page 14 of Arima Plant Hi-C 2.0 Protocol. We submitted 4.8 μg of DNA to MSGSC at BC Cancer Center. MSGSC continued the Arima Hi-C 2.0 protocol by resuspending the submitted crosslinked nuclei pellet in water and aliquoting it into 20 μl samples, the maximum input volume for the remainder of the Arima HiC protocol (document part number A160163 v00, 2020 June; Arima Genomics Inc., California, USA). MSGSC also performed the library preparation and Illumina sequencing. A single library was constructed for PE150 Illumina sequencing at ∼60 M individual reads (∼30 M read pairs) on the HiSeq platform (Illumina, California, USA). We obtained 29.3 Gb forward and 29.3 Gb reverse reads of good quality. The restriction sites used are ^GATC, G^ANTC, C^TNAG, T^TAA where ^ is the cut on the positive strand and “N” can be any of the 4 genomic bases.

### Hi-C scaffolding

Hi-C data were used to refine both assemblies, genomic pools A and B. We followed step 0 through step 4 in Arima Genomics Hi-C Mapping Pipeline: UserGuide_A160156_v02 (released May 2019). The final output of the pipeline is a single BAM file that contains the paired, 5′-filtered, and duplicate removed Hi-C reads mapped to the draft assemblies. We then used BEDTools v2.30.0 (Quinlan and Hall 2010) to convert the BAM file into a sorted BED file for use in SALSA v2.3. SALSA is a tool to scaffold long-read assemblies with Hi-C data (Ghurye *et al*. 2019; 2017). Next, we made a Hi-C file with the converst.sh script included in the SALSA documentation (Ghurye *et al*. 2019; 2017) to upload in Juicebox v1.11.08 (Durand *et al*. 2016) for visualization as a Hi-C contact map for each assembly A and B (Supplementary Fig. 6).

BUSCO v5.4.6 was used to assess the completeness of the final assemblies with “–metaeuk” (Table 1), and QUAST v5.0.2 was used to generate statistics on assemblies A and B (Table 1) (Gurevich *et al*. 2013). In assembly A, 37 out of 38 contigs are above 50,000 bp with the longest contig being 9,932,022 bp. In assembly B, 46 out of 50 contigs are above 50,000 bp with the longest contig being 10,910,790 bp. Using minimap2 v2.24 (Li 2018), assembly B was mapped against assembly A and the generated alignment was visualized as a dot plot (Fig. 3c) with dotPlotly (https://github.com/tpoorten/dotPlotly). The filtered Illumina and Nanopore data were mapped to the final polished assemblies using BWA (Li and Durbin 2010), and mapping statistics were calculated using SAMtools v1.17 (Danecek *et al*. 2021). The data quantity, depth, coverage, and mapping rates to assemblies A and B are summarized in Supplementary Table 3. Read histograms were generated for the Nanopore and Illumina data mapped to the final (polished and Hi-C scaffolded) assemblies for Genomes A and B using purge_haplotigs (Supplementary Fig. 7; https://github.com/skingan/purge_haplotigs_multiBAM; Roach *et al*. 2018).

**Table 1.** Genome statistics generated by QUAST and BUSCO for genome A and B *S. aurantia* variants.

	*S. aurantia* Genome A	*S. aurantia* Genome B	*C. reinhardtii*	*Chlamydomonas* sp*. ICE-L*	*C. priscuii*	*L. spitsbergensis*
**Total length (Mb)**	96	102	111.2	541.9	211.6	260.2
**# Scaffolds**	38	50	53	946	2,458	124
**N50 (Mb)**	5.42	6.41	8	19	0.37	3.9
**L50**	8	7	7	5	165	21
**GC %**	57.48	55.10	64	49	60.61	54.2
**BUSCO score (n:1519)**	C:87.4% [S:86.9%, D:0.5%], F:2.5%, M:10.1%	C:81.9% [S:81.2%, D:0.7%], F:3.6%, M:14.5%	C:98.9% [S:92.9%, D:6.1%], F:0.3%, M:0.7%	C:78.5% [S:67.0%, D:11.5%], F:6.6%, M:14.9%	C:87.0% [S:80.2%, D:6.8%], F:3.7%, M:9.3%	C:90.0% [S:76.8%, D:13.2%], F:3.7%, M:6.3%

The table also includes the reference values for 3 psychrophilic green alga, *Chlamydomonas* sp. ICE-L, *C. priscuii*, and *L. spitsbergensis* as well as the model temperate green alga *C. reinhardtii*. Benchmarking of Universal Single-Copy Orthologs (BUSCO): C, complete, D, duplicated; F, fragmented; M, missing; S, single.

### RNA sequencing

The green culture of *S. aurantia* was grown at 4°C with 115 μmol m^−2^ s^−1^ SunBlaster grow lights (bulb model F24TK 24W HO 6400K). In April 2023, cells were harvested using a benchtop microcentrifuge for a total pelleted volume of 35μl (Thermo Fisher), flash frozen on dry ice, and shipped on the same day. A large mass of dry ice and the sample were shipped in a Styrofoam box to Azenta US Inc., South Plainfield, New Jersey. Azenta performed Nucleic Acid Extraction, Sample QC, Illumina library preparation with PolyA selection, and standard RNA sequencing. Extraction was performed with RNeasy Plant Mini Kit (50) Cat. No./ID: 74909 (Qiagen, Hilden, Germany) and Plant RNA Isolation Aid AM9690 (Thermo Fisher). RNA sequencing was performed using 150 nucleotide PE reads with a single index. We obtained 466,411,247 reads (139,923 Mb yield), 33 Gb forward and 35 Gb reverse reads, with 94% of bases ≥ 30 Phred quality score (a logarithmic scale used to measure the accuracy of base calls based on sequencer-defined probability of error in each base call) and a mean quality score of 35.5, representing a 99.9% inferred base call accuracy.

### Mapping of RNA-Seq reads

RNA-sequencing data quality was analyzed with FastQC v0.12.0, and adaptors and low-quality bases were trimmed using Trimmomatic v0.39 (Bolger et al., 2014) and AdaptorRemoval v2 (Schubert et al., 2016). The splice-aware aligner STAR v2.5.2 (Dobin *et al*. 2013) was used to generate BAM files for gene prediction. The rate of uniquely mapped reads was 33.5% for the high GC pool and 45.6% for the low GC pool. SAMtools v1.17 was used to convert our alignment file into sorted BAM file format for use in genome annotation (Danecek *et al*. 2021).

### Repeat finding and repeat masking

Repeat Modeler v2.0.3 was used to construct a custom repeat library for *S. aurantia* using our assemblies as input (Flynn *et al*. 2020). Repeat Masker v4.1.2 was used to search for interspersed repeats and low-complexity DNA sequences using the custom *S. aurantia* repeat library; the genome was soft-masked for downstream analyses (Maja and Chen 2009). Repeat Masker v4.1.2 identified 13.25% of repeat content in genome A and 13.81% of repeat content in genome B (Maja and Chen 2009).

### Genome annotation

A combination of ab initio, homology-based, and RNA-Seq-based predictions was used for gene annotation. The automated pipeline BRAKER3 v3.0.3 (Hoff *et al*. 2019) utilizes AUGUSTUS (Stanke and Morgenstern 2005) and GeneMark-ES/EP + (Borodovsky and Lomsadze 2011; Brůna *et al*. 2020) to predict protein-coding gene structures in novel eukaryotic genomes. As input, we provided Viridiplantae proteins from SwissProt (Bairoch and Apweiler 2000) for homology-based gene predictions and an aligned BAM file of RNA sequencing reads for evidence-based gene predictions. The soft-masked assembly was used as the query. The first round of BRAKER was run de novo, and the output was used to train the gene models for AUGUSTUS and GeneMark-ES/EP + for a second round of BRAKER3 v3.0.3 with the trained models (Hoff *et al*. 2019). We assigned functional annotations from the best Basic Local Alignment Search Tool (BLAST) hit of the protein-coding genes by using BLASTP v2.2.26 (*E*-value < 1*e*–5) against the SwissProt database. InterProScan v.5.64-96.0 was used to annotate Pfam domains, which integrates predictive protein function information from partner resources (Quevillon *et al*. 2005), and we used the Pfam database of curated protein families (-appl pfam; Finn *et al*. 2014) and retrieved gene ontology (GO) terms (-goterms; Balakrishnan *et al*. 2013). GO terms were submitted to cateGOrizer, a GOTerms Classifications Counter (https://www.animalgenome.org/cgi-bin/util/gotreei), to categorize their occurrences within the predefined set of parent GO terms within the Plant GO_slim subset. We made the complete tabular output of cateGOrizer for assemblies A and B available in Supplementary Table 4 and manually identified GO terms with potential adaptive functions in assembly A to highlight in Table 2. tRNAscan-SE v.2.0.12 was used to predict tRNA genes using default parameters (Chan *et al*. 2021).

**Table 2.** Select plant GO terms of interest in genome A which may be related to snow alga adaptation to the snow.

Definition	Function	GO ID
Response to extracellular stimulus	Cellular response to starvation	GO:0009267
Lipid metabolic process	Lipid metabolic process	GO:0006629
	**Lipid biosynthesis**	GO:0008610
	Fatty acid metabolic process	GO:0006631
	Fatty acid biosynthetic process	GO:0006633
	Fatty acid beta oxidation	GO:0006635
	Fatty acid beta oxidation using	GO:0033539
	Acyl-CoA dehydrogenase	GO:0009247
	Glycolipid biosynthetic process	GO:0030259
	Lipid glycosylation	GO:0008654
	Phospholipid biosynthetic process	GO:0019288
	Isopentenyl diphosphate biosynthetic process, methylerythritol 4-phosphate pathway	
Photosynthesis	Photosystem II repair	GO:0010206
	Photosystem II stabilization	GO:0042549
	Photosystem II assembly	GO:0010207
	Photosynthesis	GO:0015979
Response to stress	Response to oxidative stress	GO:0006979
	DNA damage response	GO:0006974;GO:0000077
	Double-strand break repair via Nonhomologous end joining	GO:0006303
	Double-strand break repair	GO:0006302
	Mismatch repair	GO:0006298
	Nucleotide-excision repair	GO:0006289
	Base-excision repair	GO:0006284
	Regulation of DNA repair	GO:0006282;GO:0006281
	Homologous recombination	
	Recombinational repair	GO:0000725
	Double-strand break repair via	GO:0000724
	DNA damage checkpoint signaling	GO:0000077
Motor activity	Cytoskeletal motor activity	GO:0003774
	Microtubule motor activity	GO:0003777;GO:0008569
Other	Vitamin B6 biosynthetic process	GO:0042819

### Highly similar duplicate genes predictions

**Fig. 4.**
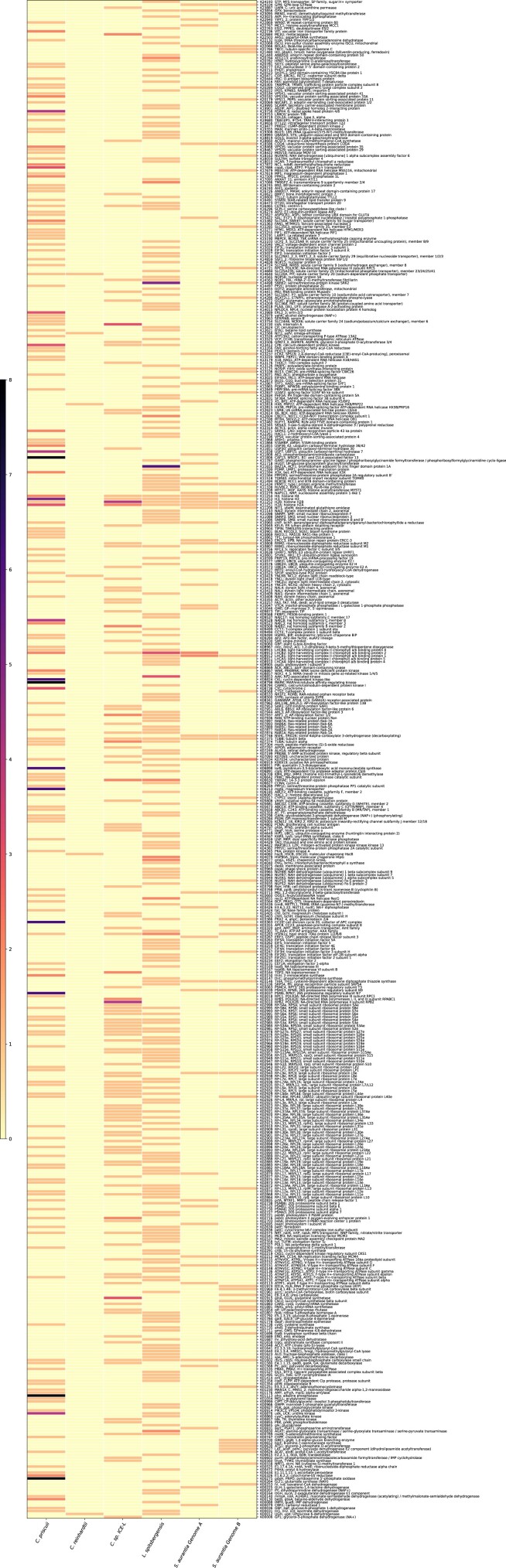
Comparative genome analyses across the chlamydomonadalean species: *C. priscuii*, *C. reinhardtii*, *C.* sp*. ICE-L*, *L. sp*i*tsbergensis*, and *S. aurantia* Genomes A and B. A heatmap of KEGG functional categories. The color scale indicates the number of HSDs, 0 does not mean the category is absent. KEGG functional categories are indicated on the side, such as carbohydrate metabolism, energy metabolism, and translation.

Predicting highly similar duplicates (HSDs) is a workflow adapted from Zhang *et al*. (2021a; 2021b). We first used our *S. aurantia* gene models to create a custom BLAST database and then conducted an all-against-all protein BLAST v2.2.26 (Altschul *et al*. 1990) search of the gene models against themselves (*E*-value cutoff 10^−5^; set -outfmt 6). With both the BLAST result (list of HSDs) and InterProScan functional descriptions, we ran the program HSDFinder.py (Zhang *et al*. 2022), which filters duplicates with near-identical protein lengths. We set the parameters for gene duplicates to be within 10 amino acids in length and 90% pairwise identities. The amino acid sequences of the HSDs were used as input for BlastKOALA for automatic KO assignment and KEGG mapping, which generates a list of gene identifier matched with a KO accession number retrieved from the KEGG database (Kanehisa *et al*. 2016). The KEGG gene list and HSD file were input to the web server HSDfinder (http://hsdfinder.com) to produce a heatmap (Fig. 4). We repeated this same protocol (Zhang *et al*. 2021b) for the recently sequenced and annotated genome *Limnomonas spitsbergensis* (Hulatt *et al*. 2024). We also incorporated the available nuclear genome data from *Chlamydomonas priscuii* (renamed from sp. UWO241; Stahl-Rommel *et al*. 2022), *Chlamydomonas* sp. ICE-L (Zhang *et al*. 2020), and *Chlamydomonas reinhardtii* (Merchant *et al*. 2007) on HSDfinder (filtering option more than 90% amino acid pairwise identity and within 10 amino acid differences) to compare between species. Complement to the heatmap, the HSDFinder web server output for assemblies A and B is available in tabular format (Supplementary Table 5).

### Comparative genomic analyses

**Fig. 5.**
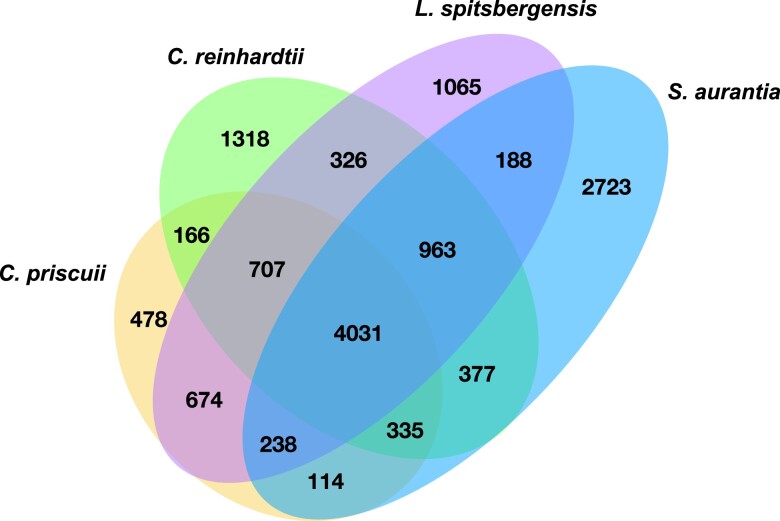
Comparative genomic analysis across the chlamydomonadalean species: *S. aurantia* (genomes A and B collectively), *C. priscuii*, *L. sp*i*tsbergensis*, and *C. reinhardtii*. Venn diagram displaying unique and shared gene families across the species. Orthologous genes searched using the program OrthoMCL.

To search for homologous genes, we compared *S. aurantia* assemblies against protein sequences from psychrophilic genomes, *C. priscuii* (Cvetkovska *et al*. 2019) and *L. spitsbergensis* (Hulatt *et al*. 2024), and a temperate genome, *C. reinhardtii* (Merchant *et al*. 2007), retrieving these reference genome annotations from the NCBI (Agarwala *et al*. 2018) and Zenodo (https://zenodo.org). We used OrthoMCL v2.0.9 to construct homologous gene clusters (orthogroups) and visualized these data in a manually curated Venn diagram (Fig. 5).

## Results and discussion

### Sequencing, genome assembly, and genome quality

We used Oxford Nanopore and Illumina to sequence the green biciliate culture of *S. aurantia*, grown from a field sample collected on Brandywine Mountain, British Columbia, Canada, in July 2019 (Raymond *et al*. 2022). After filtering for quality, Oxford Nanopore sequencing on a PromethION yielded 44.3 Gb of long-read data, and Illumina sequencing on a HiSeq yielded 13.5 Gb of forward and 13.5 Gb of reverse short-read data. We generated 2 genome assemblies of *S. aurantia*, presumed to be 2 variants present in our culture, identifiable based on a difference in GC content and difference in read depth (Fig. 3). We define genome A as the genomic pool of contigs with a higher average GC base composition of 57.5 and higher average read depth of ∼43 ×. Genome B is defined as the genomic pool of contigs with a lower average GC base composition of 55.1% and a lower average read depth of ∼ 13X. We obtained an additional 29.3 Gb of forward and 29.3 Gb of reverse Illumina Hi-C sequencing data to improve both assemblies using contact information for scaffolding. Both variant assemblies are similar when evaluated using metrics of contiguity and gene content completeness. Genome A is 96 Mb, composed of 38 scaffolds with an N50 of 5.4 Mb, capturing 87.4% of the Chlorophyta universal single-copy orthologues with BUSCO (Table 1). Genome B is 102 Mb, composed of 50 scaffolds, has an N50 of 6.4 Mb, and captured 81.9% of Chlorophyta completeness with BUSCO (Table 1).

Polishing and Hi-C scaffolding made an overall improvement to the genome assemblies by reducing the number of contigs and increasing the N50 value. The initial draft genome A was improved from 41 to 38 contigs and an increase in N50 from 3.5 to 5.4 Mb, and the initial draft genome B was improved from 58 to 50 contigs and an increase in N50 from 2.8 to 6.4 Mb (Supplementary Table 1). We mapped the Illumina PE reads and Oxford Nanopore long-reads to each final assembled genome to assess assembly accuracy. With Illumina reads, 54.5 and 54.4% mapped to genomes A and B, respectively (Supplementary Table 3). With ONT reads, 93.4 and 93.9% mapped to Genomes A and B, respectively (Supplementary Table 3). No split reads were identified, but the genomes are similar enough that many reads mapped independently to both genomes.

### Taxonomic partitioning

We expected contaminant sequences because the culture has not been grown axenically, but our approach was to include all Oxford Nanopore data in the initial assembly to create a more contiguous genome and then later remove assembled contaminant contigs. We used BlobTools (Laetsch and Blaxter 2017) as a taxonomic partitioning tool to retain only Chlorophyta data in our assembly. Although a total of 97.9% of Illumina reads mapped back to our initial genome assembly, BlobTools identified only 57.7% of these as Chlorophyta (Supplementary Fig. 3). Nearly all the contamination belonged to Pseudomonadota at 34.0% and undefined Eukaryota at 4.1%. Pseudomonadota (synonym Proteobacteria) are often one of the dominating bacteria in snow algal blooms (Supplementary Fig. 3) (Soto *et al*. 2020; Krug *et al*. 2020; Yakimovich *et al*. 2020). We only kept Chlorophyta contigs past this stage in the assembly, which removed 87 contigs and 45.8 Gb of data total.

### *Sanguina* genetic variability

With only 2 named species of *Sanguina* to date, we hypothesize that the genus contains unexplored diversity. High-throughput sequencing studies have attempted to categorize bloom composition (Brown *et al*. 2016; Lutz *et al*. 2017; Davey *et al*. 2019; Engstrom *et al*. 2020) but vary in resolution based on the marker used. We expect that genome-wide sequencing will reveal genomic variation in a bloom at the genus and species level that is not currently captured by selected markers of short regions of conserved DNA. *Sanguina* is a cosmopolitan and widespread genus with many ITS2 haplotypes reported in global comparisons (Procházková *et al*. 2019; Raymond *et al*. 2022). A recent study combining 18S rDNA gene and ITS2 amplicon sequencing suggests there are at least 2 as-of-yet undescribed *Sanguina* species (Remias *et al*. 2023). Based on the 2 *S. aurantia* variants originating from one field sample, we predict that *Sanguina* may be a genus with high genetic variability.

### Construction of GC-based genomic pools

We detected a significant amount of duplication (65.2%) in our original assembly and observed two peaks in a frequency histogram of Illumina reads mapped back to the genome assembly (Supplementary Fig. 5a and b and Table 2). We used BlobTools as a visualization tool and identified 2 variants in our assembly, which could be separated based on GC content (Fig. 3; Laetsch and Blaxter 2017). GC content had a strong correlation with read depth (Fig. 3a, Supplementary Table 2). After the separation of contigs on the basis of GC content, genome A (average GC 57.5%) revealed a single peak at ∼43-fold coverage, whereas genome B (average GC 55.1%) had a large peak at lower coverage (∼13-fold) with a small second peak at (∼43×) (Fig. 3b, Supplementary 5c and d). We suspect that the separation of contigs on the basis of GC content did not provide a clean split between variants, possibly explaining the reduced second peak in genome B as derived from genome A contigs (Fig. 3b, Supplementary 5d). However, both Genomes A and B represent highly complete and contiguous assemblies based on N50 and BUSCO scores (Table 1), showing GC content as a useful tool to navigate the potentially large genetic variability within snow algae species. These findings raise the possibility that published cold-adapted algal genomes might not be as extensively duplicated as reported.

In plants, the highest variation in GC content is in green algae with variation evident between and within species (Šmarda and Bureš 2012). Thirty-eight strains of the golden-brown alga *Synura petersenii* varied between 37.1 and 41.2% in GC content (Čertnerová and Škaloud 2020), and the marine green alga *Ostreococcus tauri* exhibits within-genome variation in GC content ranging from 30 to 77% (Toulza *et al*. 2012). The unique heterogeneity found in the *O. tauri* genome, most noticeable in 2 of the 20 chromosomes, raises the question of whether within-genome GC variation is specific to lifestyle and ecology and whether this is more common in eukaryotes than so far represented by the “models” dominating genome databases (Derelle *et al*. 2006). The DNA and GC content of an organism under stress are more likely to undergo dramatic changes, due to increased DNA repair, DNA repetition, or duplicated gene copies (Smarda and Bures, 2012). Snow algae live in a stressful environment, and a variation in GC content either between variants or within a single genome is reflective of this. We see evidence of a number of DNA repair mechanisms in *the S. aurantia* genome, including error-prone mechanisms such on nonhomologous end joining which can cause duplications, genomic expansion, and rearrangements (Table 2) (Christensen 2013; Fang *et al*. 2021a; Fang *et al*. 2021b).

### Annotation

We obtained 33 and 35 Gb forward and reverse reads of RNA-Seq data with Illumina sequencing to provide evidence-based annotation. We mapped RNA-reads back to Genomes A and B and found 33.5 and 45.6% mapped to the respective assemblies. This difference might be explained by differential growth of the two variants during the 2-year gap between genome sequencing and RNA sequencing. After two rounds of BRAKER, a total of 11,258 protein-coding genes were annotated for genome A and 12,264 protein-coding genes were annotated for genome B. With BUSCO set to protein mode, genome A was 85.9% complete and genome B was 80.9% complete. Functional annotation identified 64 and 60% of protein-coding genes in Genomes A and B with known homologs in the SwissProt protein database (Boeckmann *et al*. 2003). In Table 2, we report some of the pathways with proposed benefits to cold-adapted genomes including those involved with lipid metabolic processes, photosynthesis, response to extracellular stimulus, and stress.

Astaxanthin is the prominent secondary carotenoid responsible for the defining red pigmentation of the genus *Sanguina* (Fig. 1). The physiochemical characteristics of lipid droplets in *S. nivaloides* have been studied as a key adaptive trait to the snow environment (Ezzedine *et al*. 2023). Astaxanthin-rich lipid droplets protect the cell contents against high irradiance and oxidative stress (Ezzedine *et al*. 2023). The accumulation of other lipids including triacylglycerols and polyunsaturated fatty acids also serve a role as a carbon-dense storage form and to maintain fluidity in photosynthetic membranes at freezing temperatures (Ezzedine *et al*. 2023). Fatty acid, glycolipid, and phospholipid biosynthesis are evidenced in the *S. aurantia* genome (Table 2). In addition, we found the methylerythritol 4-phosphate (MEP) pathway for synthesis of isopentenyl pyrophosphate, a precursor for astaxanthin synthesis, in the *S. aurantia* genome (Lichtenthaler 1987). This is the same pathway used by the freshwater green microalga *Haematococcus pluvialis* for astaxanthin biosynthesis (Luo *et al*. 2019; Nair *et al*. 2023).

Photosystem II (PSII) is susceptible to photoinhibition in excess light, but as first shown in *C. reinhardtii*, turnover of the chloroplast-encoded D1 protein can play an important role in PSII recovery (Wettern and Ohad 1984; Niyogi 2009). D1 undergoes oxidative damage during photoinhibition, a process which may be exacerbated in a low nutrient environment when the rate of photosynthesis is reduced (Grossman 2000). In Table 2, we report PSII repair, stabilization, and assembly gene ontology terms evident in the *S. aurantia* genome involved with proteolysis of the damaged D1 protein and re-assembly of a new D1 subunit in the PSII following photoinhibition.

Without RNA-Seq under different conditions, we can only note the presence or absence of certain molecular functions but not whether they are up- or down-regulated, for example, when under cold stress. We did not identify any genes encoding ice-binding proteins (IBP). This may relate to the sensitivity of *Sanguina* to freezing conditions. In *C.* sp. ICE-L (Zhang *et al*. 2020) and *C. priscuii* (Zhang *et al*. 2021a, 2021b), IBP were acquired via horizontal gene transfer. Some possible reasons they are not present in the *S. aurantia* genome are that they were removed with bacterial contaminant sequences or that they are absent in snow-inhabiting species compared to those that live directly on the ice. The morphology of *S. aurantia* when cultivated under the relatively low light conditions of our incubator is ciliated (Raymond *et al*. 2022). Thus, we were not surprised to identify genes for ciliary proteins including dynein's and kinesins (Table 2).

### Comparative genomics

To date, there are 117 chlorophyte genomes publicly available on the NCBI (as of 2024 June 11; NCBI). *C. reinhardtii* was one of the earliest Eukaryotic genomes to be sequenced and has served as a model for chlorophytes (Grossman *et al*. 2003; Merchant *et al*. 2007). By generating a more complete database of green algae, we can use comparative genomics as a tool to expand our knowledge of Viridiplantae evolution. The ecological diversity of green algae gives insight to its potential range of functional capabilities, yet, unculturable species, particularly those living in extreme environments, have so far been underrepresented in genomic databases.

Green strains identified as *C. nivalis*—a species name attributed to what we now know to be a polyphyletic collection of diverse genera of microalgae—have been used for studies to describe snow algal adaptation (Hader and Hader 1989; Hounslow *et al*. 2021; Lu *et al*. 2012; Peng *et al*. 2021). Whether any of these studies are relevant to *Sanguina* is unknown. Indeed, some of the algae used in these studies might not even been snow algae as they are grown in the UTEX culture collection at temperatures incompatible with the growth of snow algae (UTEX 2824; Lu *et al*. 2013; 2012; Peng *et al*. 2021; Zheng *et al*. 2020). It was not until the successful culturing of *S. aurantia* in 2019 that a culture conspecific with a red-jeweled species has been available for sequencing (Raymond *et al*. 2022). We set out to identify the shared and unique characteristics of snow algae when compared with polar and temperate alga.

The genomes of 3 cold-adapted *Chlamydomonas* species were recently reported: *C.* sp. *ICE-L* which lives on polar sea ice in Antarctica (Zhang *et al*. 2020), *C. priscuii* which resides in the permanently ice-covered Lake Bonney in McMurdo Dry Valleys, Antarctica (Cvetkovska *et al*. 2019; Stahl-Rommel *et al*. 2022), and *L. spitsbergensis* isolated from a sample of melting snow on Spitsbergen, Svalbard (Hulatt *et al*. 2024). All cold-adapted genomes displayed characteristics unique from the temperate model green alga *C. reinhardtii*, which lives in soil habitats. Most notably, all cold genomes *C. priscuii*, 212 Mb, *C.* sp. *ICE-L*, 542 MB, and *L. spitsbergensis*, 260.2 Mb, were larger than *C. reinhardtii*, 110 Mb, and had a larger amount of gene duplication (Fig. 4; Cvetkovska *et al*. 2019; Hulatt *et al*. 2024; Merchant *et al*. 2007; Zhang *et al*. 2020). The genome of *S. aurantia* is smaller than any of these at 96 Mb for one variant and 102 Mb for the other (Table 1).

The *S. aurantia* genome has the lowest number of annotated protein-coding genes, 11,258 (A) and 12,264 (B) compared with 15,143 in *C. reinhardtii* (Merchant *et al*. 2007), 19,870 in *C.* sp*. ICE-L* (Zhang *et al*. 2020), 16,325 in *C. priscuii* (Zhang *et al*. 2021a, 2021b), and 18,277 in *L. spitsbergensis* (Hulatt *et al*. 2024). The contiguity and completeness of *S. aurantia* are high relative to the references, with the fewest scaffolds and N50 values of 5.4 Mb (genome A) and 6.4 Mb (genome B), comparable to the model for green alga *C. reinhardtii*, with an 8 Mb N50 (Table 1) (Blaby *et al*. 2014; Merchant *et al*. 2007). We compared the annotated gene sets of *S. aurantia* collectively against the temperate alga *C. reinhardtii* and cold-adapted algae *C. priscuii* and *L. spitsbergensis*, by clustering orthologous groups (Fig. 5). We identified 4,031 gene families shared among all 4 genomes, 238 gene families shared by the 3 cold-adapted genomes, and 2,723 gene families unique to *S. aurantia* (Fig. 5).

We align and compare *S. aurantia* Genomes A and B in a dot plot (Fig. 3c) (Supek *et al*. 2011). Differences between variants have been seen in the diatom *Fragilariopsis cylindrus*, possibly involved in adaptation to the fluctuating environment of the Southern Ocean (Mock *et al*. 2017). Another example exists in the bloom-forming alga *Prymnesium parvum*, where 15 strains revealed within-species diversity and variable gene families (Wisecaver *et al*. 2023). Notably, *P. parvum* can survive in a wide range of aquatic habitats from eutrophic to alkaline to brackish and strains were divergent with some hybrids that retained 2 haplotypes (Wisecaver *et al*. 2023). These studies suggest that the differences between *S. aurantia* genome variants may reflect true genomic variability caused by the snow environment (Fig. 3).

The cold-adapted genomes of *C.* sp. ICE-L and *C. priscuii* are reported to have high levels of gene duplication. Therefore, we searched for HSDs in our genome assembly to identify how many genes were represented 2 or more times within each assembly. To identify multicopy genes, we used a BLAST search of *S. aurantia* gene models against themselves (*E*-value cutoff 10^−5^), filtering for pairwise amino acid identities > 90% within 10 amino acids in protein length. We identified 161 (genome A) and 176 (genome B) putative duplicates with one or more copies (Fig. 4). Consistent with previous cold-adapted genomes, *S. aurantia* has a greater duplication than the temperate *C. reinhardtii* which only has 54 duplicates. Yet, *S. aurantia* has fewer than the extensively duplicated *C. priscuii* with 336 duplicates and *C.* sp*. ICE-L* with 265 duplicates (Zhang *et al*. 2021a, 2021b), when implementing the same similarity threshold.

The protein sequences of HSDs were searched against the KEGG functional database (Supplementary Table 5), with many duplicated genes involved in the metabolism of carbohydrates, lipids, energy, nucleotides, amino acids, and vitamins (Supplementary Table 5). Some shared HSDs across the cold-adapted genomes of *C.* sp*. ICE-L*, *C. priscuii*, *L. spitsbergensis*, and *S. aurantia* are involved with protein translation, DNA packaging, and photosynthesis including histones, light-harvesting complexes, and ribosomal proteins. The expression of genes is dependent on the regulation of chromatin states (active or repressed), transcription, and translation. In a cold-stressed environment, chromatin dynamics affect genome stability and the ability to regulate gene expression to respond to unfavorable environmental conditions; therefore, it is unsurprising for these genes to be duplicated in the phenotypically plastic genomes of *S. aurantia*, *C.* sp*. ICE-L*, *L. spitsbergensis*, and *C. priscuii* (Kim 2021; Park *et al*. 2018).

### Limitations of the study

It is likely that the 2 variants of *S. aurantia* are not cleanly delineated by GC content and thus, while we think each genome is largely representative of a single variant supported by different sequencing coverages, there may be a mixed set of *S. aurantia* contigs in each assembly. This may cause an overrepresentation of certain genes annotated in one assembly and a lack of certain genes in the other. As a measure of control, the BUSCO completeness score of each genome was calculated using both “–genome” and “–transcriptome” mode with the assembly and annotated coding sequences, respectively. Comparing the results between genome A, 87.4% complete in “–genome” mode and 85.9% complete in “—transcriptome” mode, and genome B, 81.9% complete in “–genome” mode and 80.9% complete in “—transcriptome” mode, indicative of an even divide of genomic content. When testing for split reads using BWA with the “bwa mem -m” flag, we do not find any reads that align partially or to 2 locations, indicating that each assembly is contiguous and that there are no chimeric contigs.

Although GC content is a common approach used to distinguish species and genomes (Karimi *et al*. 2018), it is important to consider the GC bias introduced by next-generation sequencing (Benjamini and Speed 2012; Chen *et al*. 2013). GC-poor regions typically have a lower sequencing coverage than GC-rich regions (Browne *et al*. 2020; Delahaye and Nicolas 2021). This bias could contribute to the direct relationship between GC content and coverage in our 2 genomes, with genome B having lower GC content and lower coverage. However, GC bias is more prominent in Illumina data than Oxford Nanopore (Goldstein *et al*. 2019; Sevim *et al*. 2019) and does not affect Oxford Nanopore pipelines (Browne *et al*. 2020). More likely, the higher GC variant was present at higher density than the low GC variant in the culture at the time of sequencing. We also note that the polishing steps changed the average GC composition of genome B (Supplementary Table 1) and is a limitation of having metagenomic data from a single culture mapping to both of the 2 variants.

## Conclusion

Snow algae provide an opportunity to study the diversification of green algae adapted to a cold environment. We have made available DNA and RNA sequencing data of the cosmopolitan and abundant red-snow-populating genus *Sanguina*, to facilitate future comparative genomic and transcriptomic analyses. We present the genome assemblies of 2 *S. aurantia* variants, distinguishable based on GC content. Genome A is 96 Mb with 38 scaffolds and 5.4 Mb N50 with a GC content of 57.5%; genome B is 102 Mb with 50 scaffolds and 6.4 Mb N50 with a GC content of 55.1%. Sequencing reads originated from a culture of *S. aurantia* grown from a red snow field sample. We outline a reproducible bioinformatic pipeline to assemble highly contiguous and complete green algal genomes. Many nonmodel species are limited by the lack of a culture or by complex data with no references, we hope for this pipeline to contribute a novel perspective on approaching de novo assemblies.

We utilized Oxford Nanopore and Illumina Technology in a hybrid approach to assemble, polish and scaffold using Hi-C links, 2 high-quality genomes with BUSCO completeness scores of 87.4 and 81.9% (Table 1). Our data suggest the presence of high genomic variability within *S. aurantia*. We acknowledge the alternate possibility of GC variation and high duplication within a single genome, suggesting that the 2 sets of annotation data presented may be considered jointly for *S. aurantia*. The nuclear genome structure and content of a snow algae presented in this study are aimed to set the foundation for future green algal comparative research and to provide reference for related Chlorophyte assemblies.

## Supplementary Material

jkae181_Supplementary_Data

## Data Availability

This Whole Genome Shotgun project has been deposited at DDBJ/ENA/GenBank under the accession JBBJLO000000000 for genome A and JBBFKH000000000 for genome B, processed as metagenome assembled genomes (MAGs) from the same environmental sample. The physical green algal culture (derived from red snow) has a BioSample ID SAMN40583292, and all sequencing data are linked to this environmental sample on the sequence read archive (SRA; NCBI): Oxford Nanopore (SRR28420822), Illumina WGS (SRR28420823), Hi-C (SRR28420821), and RNA sequencing (SRR28420820). All data are available under the BioProject PRJNA1085394, with the Bio Sample accession SAMM40302126 assigned to genome A and BioSample accession SAMM40302127 assigned to genome B, and raw reads derived from the green biciliate culture under BioSample SAMN40583292. A sample of dead biomass of the *S. aurantia* culture (CCCryo 569–24) is available at the Culture Collection of Cryophilic Algae (CCCryo; Potsdam-Golm, Germany). Live cells are currently available from our labs in Canada and France. We hope they will soon also be available from CCCryo. Assembly scripts are available on GitHub https://github.com/breannar/Genome_MS. Supplemental material available at G3 online.
