# Supplementary material for: Snow alga Sanguina aurantia as revealed through de novo genome assembly and annotation
Source: G3 (Bethesda). 2024 Aug 2;14(10):jkae181. doi: 10.1093/g3journal/jkae181 (PMC11457085; doi:10.1093/g3journal/jkae181)
Supplement: jkae181_Supplementary_Data [file jkae181_supplementary_data.zip › Figure_S1_G3-2024-405201.pdf]

PFGE QC – 210401MDJA
1% 0.5X TBE; 22 hours; 100ng
Lynne Quarmby DNA samples

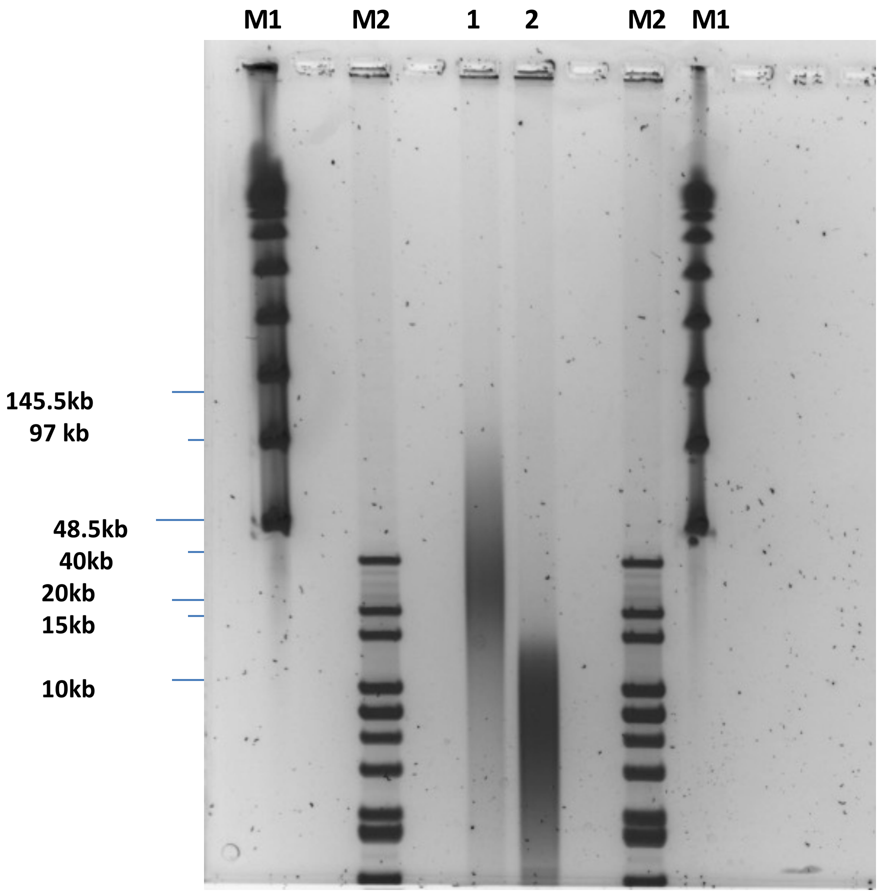

M1 – Lambda PFG Ladder
M2 – 1kb Extension Ladder
1 – F46211-BC3_Feb18_2021_purified
2 – F46212-BC1_Mar15_2021_bead
