# Supplementary material for: Snow alga Sanguina aurantia as revealed through de novo genome assembly and annotation
Source: G3 (Bethesda). 2024 Aug 2;14(10):jkae181. doi: 10.1093/g3journal/jkae181 (PMC11457085; doi:10.1093/g3journal/jkae181)
Supplement: jkae181_Supplementary_Data [file jkae181_supplementary_data.zip › Supplemental_Material_Legends_G3-2024-405201.docx]

**Supplemental Figure Captions**

Figure S1. Two gDNA samples, lane 1 and 2, run on a pulse-field gel electrophoresis (PFGE QC) on 1% 0.5X TBE for 22 hours at 100ng, sample processed by Canada’s Michael Smith Genome Sciences Centre at BC Cancer. The extraction method with bead beating removed shown in lane 1 has a higher molecular weight, indicative of a less fragmented gDNA sample. The sample in lane 1 was selected for both Oxford Nanopore and Illumina sequencing.

Figure S2. GenomeScope was used to estimate the overall characteristics of the *S. aurantia* genome using Illumina short-reads as input. This includes genome size, heterozygosity rate and repeat content.

Figure S3. Blobtools was used to visualize the quality of the initial assembly. A Blast-search output and alignment file of mapped Illumina reads were used as input. The bar chart on the left displays that 97.9% of Illumina short-reads mapped back to the initial genome assembly. Of those, only 57.7% identified as Chlorophyta and the remainder of the contigs were removed. Phylum assignment is based on BLAST result: ‘Eukaryota-undef’ refers to an undefined Eukaryote.

Figure S4. Blobtools was used to visualize the quality of the initial assembly. A Blast-search output and alignment file of mapped Illumina reads were used as input. The two-dimensional scatter plot is decorated with coverage and GC histograms. Contigs are represented by circles in the scatter plot, with circle diameter proportional to sequence length and all are coloured by taxonomic affiliation. The generated blobplot displays Chorophyta in blue and Pseudomonadota bacterial contaminants in orange. Phylum assignment is based on BLAST result: ‘Eukaryota-undef’ refers to an undefined Eukaryote.

Figure S5. Read-depth histogram of Illumina reads mapped to a) the initial flye assembly with all reads b) after taxonomic partitioning using BlobTools and only Chlorophyta contigs kept in the assembly c) Genome A before polishing and scaffolding and d) Genome B before polishing and scaffolding.

Figure S6. Following the Arima HiC mapping protocol, a HiC contact map was generated with SALSA and visualized in juicer. Each axes square represents the scaffolds in the order they appear in the assembly. The contact matrix derived from HiC data can be interpreted by regions of the genome with a greater interaction as having a darker red color. a) genome A and b) genome B of *S. aurantia.*

Figure S7. Histogram of ab) Nanopore reads mapped back to the final polished and scaffolded assemblies of a) genome A and b) genome B and cd) Illumina reads mapped back to the final assemblies after polishing and Hi-C scaffolding of c) genome A and d) genome B.

**Supplemental Tables**

Table S1. Genome statistics generated by QUAST and BUSCO for assembly steps starting with the a) initial assembly b) contaminants removed c) draft of genome A and d) draft of genome B. Benchmarking of Universal Single-Copy Orthologs (BUSCO): C, complete; S, single, D, duplicated; F, fragmented; M, missing.

Table S2. Illumina reads mapped to the initial assembly and analyzed with BlobTools. Length, GC content, coverage, and taxonomic assignment for each contig in the initial assembly. Contigs were split into Genome A with GC > 0.55 and into Genome B with GC < 0.55 and only contigs assigned as Chlorophyta were kept. The contigs are organized from highest to lowest GC content. Phylum assignment is based on BLAST result: ‘Eukaryota-undef’ refers to an undefined Eukaryote.

Table S3. Mapping quality of Nanopore and Illumina data to Genome A and B after polishing and scaffolding, BWA was used for mapping and SAMtools was used to generate these statistics.

Table S4. CateGOrizer output used to count and cluster Genome A and B GO term datasets in terms of the GO classes they represent, based on the GO slim plant subset database.

Table S5. HSDs of *S. aurantia* genome A and B searched against the KEGG database. HSDs_num ‘1’ indicates the presence of one duplicate copy.
