# Supplementary material for: Snow alga Sanguina aurantia as revealed through de novo genome assembly and annotation
Source: G3 (Bethesda). 2024 Aug 2;14(10):jkae181. doi: 10.1093/g3journal/jkae181 (PMC11457085; doi:10.1093/g3journal/jkae181)
Supplement: jkae181_Supplementary_Data [file jkae181_supplementary_data.zip › Table_S1_G3-2024-405201.docx]

|  | **A) Initial assembly with all nanopore reads** | **B) Initial assembly with contaminant contigs removed** | **C) ‘Clean’ nanopore assembly with contigs separated into Genome A, before polishing and scaffolding** | **D) ‘Clean’ nanopore assembly with contigs separated into Genome B, before polishing and scaffolding** |
| --- | --- | --- | --- | --- |
| **Total length (Mb)** | 248.71 | 202.88 | 96.65 | 106.23 |
| **# Scaffolds** | 186 | 99 | 41 | 58 |
| **N50 (Mb)** | 3.36 | 3.17 | 3.6 | 2.83 |
| **L50** | 27 | 22 | 11 | 12 |
| **GC %** | 55.23 | 54.87 | 57.49 | 52.48 |
| **BUSCO score (n:1519)** | C:92.8%[S:27.6%,D:65.2%],F:2.2M:5.0% | C:91.6%[S:34.3%,D:57.3%],F:2.5%,M:5.9% | C:84.5%[S:84.0%,D:0.5%],F:3.5%,M:12.0% | C:82.6%[S:81.9%,D:0.7%],F:3.6%,M:13.8% |
