# Supplementary material for: Snow alga Sanguina aurantia as revealed through de novo genome assembly and annotation
Source: G3 (Bethesda). 2024 Aug 2;14(10):jkae181. doi: 10.1093/g3journal/jkae181 (PMC11457085; doi:10.1093/g3journal/jkae181)
Supplement: jkae181_Supplementary_Data [file jkae181_supplementary_data.zip › Table_S2_G3-2024-405201.docx]

| **Contig ID** | **Length** | **GC** | **Coverage** | **Phylum** |
| --- | --- | --- | --- | --- |
| contig_104 | 1965 | 0.8402 | 0 | no-hit |
| contig_131 | 1026 | 0.6657 | 274.8251 | no-hit |
| contig_285 | 1039462 | 0.647 | 18.9588 | Pseudomonadota |
| contig_225 | 4618151 | 0.6435 | 54.7829 | Pseudomonadota |
| contig_9 | 8367 | 0.638 | 17.0845 | no-hit |
| contig_304 | 3631086 | 0.6317 | 23.8051 | Pseudomonadota |
| contig_167 | 4312186 | 0.6294 | 515.019 | Pseudomonadota |
| contig_284 | 878224 | 0.6294 | 18.5475 | Pseudomonadota |
| contig_83 | 4412628 | 0.6115 | 66.8859 | Pseudomonadota |
| contig_276 | 4814 | 0.5999 | 17.974 | Chlorophyta |
| contig_17 | 1010575 | 0.5977 | 40.971 | Chlorophyta |
| contig_295 | 55140 | 0.5958 | 179.1064 | Pseudomonadota |
| contig_117 | 8367 | 0.5933 | 14.5445 | no-hit |
| contig_175 | 670011 | 0.592 | 54.7132 | Chlorophyta |
| contig_298 | 1190353 | 0.5918 | 42.6432 | Chlorophyta |
| contig_18 | 366746 | 0.5915 | 15.2579 | Pseudomonadota |
| contig_58 | 56564 | 0.5886 | 54.1952 | no-hit |
| contig_184 | 981719 | 0.5877 | 35.8498 | Chlorophyta |
| contig_205 | 8172 | 0.5875 | 6.5944 | no-hit |
| contig_192 | 356496 | 0.5869 | 40.1234 | Chlorophyta |
| contig_146 | 844481 | 0.5856 | 58.5297 | Chlorophyta |
| contig_54 | 747861 | 0.5854 | 61.0521 | Chlorophyta |
| contig_244 | 31040 | 0.5846 | 10.2334 | no-hit |
| contig_278 | 2004003 | 0.5844 | 37.1358 | Chlorophyta |
| contig_47 | 3171003 | 0.5828 | 43.318 | Chlorophyta |
| contig_195 | 112349 | 0.582 | 37.1928 | Chlorophyta |
| contig_49 | 15753 | 0.5801 | 29.8887 | no-hit |
| contig_69 | 10162 | 0.5799 | 8.5396 | no-hit |
| contig_113 | 1078611 | 0.5798 | 42.112 | Chlorophyta |
| contig_48 | 2442068 | 0.5796 | 44.0044 | Chlorophyta |
| contig_193 | 5611 | 0.5792 | 6.5381 | no-hit |
| contig_85 | 2744692 | 0.579 | 46.7493 | Chlorophyta |
| contig_189 | 2153608 | 0.5783 | 41.779 | Chlorophyta |
| contig_290 | 3885686 | 0.578 | 38.73 | Chlorophyta |
| contig_46 | 1408437 | 0.578 | 40.5079 | Chlorophyta |
| contig_52 | 2895037 | 0.5773 | 38.6632 | Chlorophyta |
| contig_116 | 2452954 | 0.5769 | 43.1274 | Chlorophyta |
| contig_144 | 2021564 | 0.5765 | 39.7469 | Chlorophyta |
| contig_156 | 2745423 | 0.5765 | 42.1308 | Chlorophyta |
| contig_37 | 392627 | 0.5764 | 57.3887 | Chlorophyta |
| contig_44 | 2135968 | 0.5763 | 41.7452 | Chlorophyta |
| contig_119 | 2112523 | 0.5761 | 38.475 | Arthropoda |
| contig_253 | 232550 | 0.5759 | 40.7098 | no-hit |
| contig_55 | 4502136 | 0.5756 | 39.9135 | Chlorophyta |
| contig_227 | 2769588 | 0.5753 | 43.1835 | Chlorophyta |
| contig_268 | 4528 | 0.5751 | 21.903 | no-hit |
| contig_95 | 1820648 | 0.575 | 38.8253 | Chlorophyta |
| contig_94 | 3533399 | 0.5749 | 39.8614 | Chlorophyta |
| contig_233 | 3467596 | 0.574 | 42.9348 | Chlorophyta |
| contig_212 | 2658099 | 0.5731 | 39.1164 | Eukaryota-undef |
| contig_293 | 19864 | 0.573 | 507.1181 | no-hit |
| contig_177 | 4554605 | 0.5725 | 39.9413 | Chlorophyta |
| contig_31 | 6648492 | 0.5718 | 39.965 | Chlorophyta |
| contig_294 | 3461857 | 0.5709 | 39.6768 | Chlorophyta |
| contig_237 | 1908300 | 0.5707 | 40.0527 | Chlorophyta |
| contig_86 | 4522608 | 0.5705 | 45.5271 | Chlorophyta |
| contig_262 | 7759 | 0.57 | 10.2388 | no-hit |
| contig_165 | 3444196 | 0.5696 | 40.8596 | Chlorophyta |
| contig_45 | 5716752 | 0.5693 | 39.6552 | Chlorophyta |
| contig_232 | 5459498 | 0.569 | 40.2561 | Chlorophyta |
| contig_299 | 5288992 | 0.569 | 40.6394 | Chlorophyta |
| contig_215 | 223055 | 0.5686 | 55.8162 | Chlorophyta |
| contig_93 | 905265 | 0.5658 | 40.9113 | Chlorophyta |
| contig_6 | 705075 | 0.564 | 81.0698 | Chlorophyta |
| contig_75 | 42537 | 0.5633 | 15.6576 | no-hit |
| contig_110 | 31842 | 0.562 | 7.2424 | no-hit |
| contig_305 | 10138 | 0.561 | 303.5747 | no-hit |
| contig_271 | 5833 | 0.5589 | 3.6001 | no-hit |
| contig_112 | 67074 | 0.5542 | 12.5079 | no-hit |
| contig_161 | 9006 | 0.553 | 27.8959 | no-hit |
| contig_82 | 35089 | 0.5519 | 10.0872 | Pseudomonadota |
| contig_100 | 1090 | 0.5505 | 233.8526 | no-hit |
| contig_229 | 268692 | 0.5484 | 38.2122 | Chlorophyta |
| contig_245 | 49498 | 0.5469 | 11.8251 | Chlorophyta |
| contig_220 | 257853 | 0.5455 | 20.2855 | Streptophyta |
| contig_96 | 6540 | 0.5422 | 19.7682 | no-hit |
| contig_302 | 78787 | 0.5394 | 8.1051 | no-hit |
| contig_241 | 81137 | 0.5384 | 17.7426 | no-hit |
| contig_257 | 19397 | 0.5368 | 6.7173 | no-hit |
| contig_246 | 21124 | 0.5356 | 10.953 | no-hit |
| contig_136 | 5938965 | 0.5353 | 14.3664 | Chlorophyta |
| contig_21 | 291525 | 0.5347 | 18.9444 | no-hit |
| contig_256 | 11327 | 0.5345 | 4.3332 | no-hit |
| contig_7 | 997206 | 0.5327 | 28.2376 | Chlorophyta |
| contig_57 | 162125 | 0.5323 | 15.7083 | Chlorophyta |
| contig_160 | 6014 | 0.5321 | 24.2947 | no-hit |
| contig_289 | 1444321 | 0.532 | 14.1975 | Chlorophyta |
| contig_186 | 1292932 | 0.5299 | 12.1034 | Chlorophyta |
| contig_172 | 719482 | 0.5298 | 13.2018 | Chlorophyta |
| contig_122 | 2589499 | 0.5294 | 14.7883 | Chlorophyta |
| contig_11 | 846381 | 0.5289 | 16.0869 | Chlorophyta |
| contig_129 | 3091822 | 0.5289 | 13.9679 | Chlorophyta |
| contig_214 | 590 | 0.5288 | 195.5404 | no-hit |
| contig_38 | 813343 | 0.5284 | 16.4035 | Chlorophyta |
| contig_76 | 756083 | 0.5284 | 17.3286 | Chlorophyta |
| contig_125 | 979610 | 0.5283 | 12.5331 | Chlorophyta |
| contig_145 | 3442574 | 0.5283 | 14.0017 | Porifera |
| contig_181 | 2233509 | 0.5279 | 13.7364 | Chlorophyta |
| contig_279 | 336657 | 0.5278 | 11.4098 | Chlorophyta |
| contig_42 | 2068983 | 0.5275 | 13.536 | Chlorophyta |
| contig_222 | 802929 | 0.5273 | 12.5067 | Chlorophyta |
| contig_8 | 1593510 | 0.5273 | 49.3473 | Chlorophyta |
| contig_149 | 100056 | 0.527 | 55.3865 | no-hit |
| contig_70 | 4583263 | 0.5268 | 14.7966 | Chlorophyta |
| contig_101 | 2094370 | 0.5257 | 13.4953 | Chlorophyta |
| contig_273 | 2832784 | 0.5255 | 13.3188 | Chlorophyta |
| contig_35 | 7185341 | 0.5253 | 14.2983 | Chlorophyta |
| contig_264 | 2317712 | 0.5252 | 12.7982 | Pseudomonadota |
| contig_102 | 5008844 | 0.525 | 16.5457 | Chlorophyta |
| contig_126 | 1027903 | 0.525 | 13.7991 | Chlorophyta |
| contig_249 | 3227735 | 0.5249 | 14.1917 | Chlorophyta |
| contig_121 | 4624540 | 0.5248 | 16.8682 | Chlorophyta |
| contig_174 | 2837752 | 0.5248 | 15.9268 | Chlorophyta |
| contig_179 | 1708324 | 0.5247 | 21.4576 | Chlorophyta |
| contig_270 | 2273916 | 0.5244 | 15.3271 | Chlorophyta |
| contig_114 | 1417199 | 0.5241 | 14.4918 | Chlorophyta |
| contig_178 | 3367710 | 0.5241 | 14.4349 | Chlorophyta |
| contig_170 | 2487101 | 0.524 | 13.3551 | Chlorophyta |
| contig_27 | 898176 | 0.5239 | 14.7347 | Chlorophyta |
| contig_274 | 1727525 | 0.5239 | 33.9552 | Chlorophyta |
| contig_217 | 1432 | 0.5237 | 46.7311 | no-hit |
| contig_25 | 2533373 | 0.5234 | 16.9582 | Chlorophyta |
| contig_180 | 2479125 | 0.5232 | 13.2887 | Chlorophyta |
| contig_153 | 3172453 | 0.5231 | 13.1567 | Chlorophyta |
| contig_43 | 302789 | 0.5231 | 11.467 | Chlorophyta |
| contig_216 | 1499 | 0.523 | 74.179 | no-hit |
| contig_300 | 3719040 | 0.5226 | 13.7144 | Chlorophyta |
| contig_240 | 1909453 | 0.5223 | 14.3467 | Chlorophyta |
| contig_301 | 944248 | 0.5219 | 13.8162 | Chlorophyta |
| contig_288 | 859786 | 0.5218 | 11.6439 | Chlorophyta |
| contig_13 | 7480149 | 0.5216 | 16.4686 | Chlorophyta |
| contig_166 | 2198757 | 0.5215 | 13.5964 | Chlorophyta |
| contig_108 | 2562129 | 0.5213 | 13.7934 | Chlorophyta |
| contig_15 | 1546282 | 0.5206 | 12.6274 | Eukaryota-undef |
| contig_242 | 1022469 | 0.5202 | 13.4149 | Chlorophyta |
| contig_80 | 20371 | 0.5199 | 13.8852 | Pseudomonadota |
| contig_24 | 395433 | 0.5198 | 12.6666 | Chlorophyta |
| contig_213 | 8415 | 0.5194 | 21.7524 | no-hit |
| contig_269 | 8337 | 0.519 | 5.2804 | no-hit |
| contig_265 | 26574 | 0.5189 | 9.435 | no-hit |
| contig_224 | 7359 | 0.5181 | 3.5411 | no-hit |
| contig_22 | 247220 | 0.5167 | 13.0697 | Chlorophyta |
| contig_134 | 3339 | 0.5163 | 30.4602 | no-hit |
| contig_14 | 1150584 | 0.5159 | 15.0092 | Chlorophyta |
| contig_308 | 193951 | 0.5157 | 77.1955 | Pseudomonadota |
| contig_32 | 5087187 | 0.515 | 19.795 | Pseudomonadota |
| contig_81 | 11895 | 0.5142 | 14.0379 | Pseudomonadota |
| contig_188 | 664346 | 0.5141 | 12.4165 | Chlorophyta |
| contig_182 | 8387 | 0.5126 | 6.5158 | no-hit |
| contig_228 | 52826 | 0.5104 | 60.7145 | no-hit |
| contig_150 | 375867 | 0.5087 | 11.4531 | no-hit |
| contig_187 | 14852 | 0.5076 | 15.7658 | Pseudomonadota |
| contig_123 | 22005 | 0.5068 | 14.688 | no-hit |
| contig_164 | 211958 | 0.5068 | 12.2032 | Chordata |
| contig_210 | 11039 | 0.5057 | 8.1935 | no-hit |
| contig_263 | 432195 | 0.5056 | 21.7735 | Pseudomonadota |
| contig_219 | 48521 | 0.5053 | 9.0024 | no-hit |
| contig_72 | 31476 | 0.5028 | 6.792 | Pseudomonadota |
| contig_230 | 11344 | 0.5023 | 30.3094 | no-hit |
| contig_26 | 132968 | 0.5006 | 11.8293 | no-hit |
| contig_231 | 11637 | 0.4999 | 15.5667 | no-hit |
| contig_155 | 9394 | 0.4997 | 6.1136 | no-hit |
| contig_56 | 256441 | 0.4974 | 12.4182 | no-hit |
| contig_223 | 8368 | 0.4968 | 6.5458 | no-hit |
| contig_33 | 5300247 | 0.495 | 86.8075 | Pseudomonadota |
| contig_297 | 45647 | 0.4886 | 13.6818 | Pseudomonadota |
| contig_303 | 36021 | 0.4868 | 7.7718 | Chordata |
| contig_307 | 12235 | 0.4848 | 6.3586 | no-hit |
| contig_218 | 52358 | 0.4773 | 11.4667 | no-hit |
| contig_62 | 5664 | 0.4732 | 872.5078 | Eukaryota-undef |
| contig_59 | 31211 | 0.4669 | 10.2259 | no-hit |
| contig_147 | 3417 | 0.4603 | 8.848 | no-hit |
| contig_201 | 44284 | 0.4603 | 8.644 | no-hit |
| contig_73 | 4661 | 0.4336 | 60.9028 | Pseudomonadota |
| contig_60 | 134477 | 0.4262 | 440.2815 | Chlorophyta |
| contig_61 | 134438 | 0.4259 | 357.1982 | Chlorophyta |
| contig_291 | 44423 | 0.4099 | 810.1292 | Chlorophyta |
| contig_292 | 86784 | 0.4093 | 379.3394 | Chlorophyta |
| contig_66 | 86807 | 0.4093 | 460.0545 | Chlorophyta |
| contig_78 | 359786 | 0.4092 | 835.2134 | Eukaryota-undef |
| contig_63 | 71069 | 0.4084 | 843.0593 | Chlorophyta |
| contig_64 | 20360 | 0.4083 | 401.4229 | no-hit |
| contig_65 | 20372 | 0.4082 | 398.5846 | no-hit |
| contig_248 | 3896 | 0.4025 | 10.9097 | no-hit |
| contig_296 | 4788 | 0.3686 | 357.5473 | Chlorophyta |
| contig_79 | 9349 | 0.3656 | 936.9375 | Chlorophyta |
