# Supplementary material for: Snow alga Sanguina aurantia as revealed through de novo genome assembly and annotation
Source: G3 (Bethesda). 2024 Aug 2;14(10):jkae181. doi: 10.1093/g3journal/jkae181 (PMC11457085; doi:10.1093/g3journal/jkae181)
Supplement: jkae181_Supplementary_Data [file jkae181_supplementary_data.zip › Table_S3_G3-2024-405201.docx]

|  | **Genome** | **A** | **Genome** | **B** |
| --- | --- | --- | --- | --- |
|  | **Illumina** | **Nanopore** | **Illumina** | **Nanopore** |
| **Data used in assembly** | 27 Gb | 44.3 Gb | 27 Gb | 44.3 Gb |
| **Mean read depth** | 53.6 X | 149.0 X | 49.3 X | 150.2 X |
| **Breadth of coverage** | 99.2 % | 100.0 % | 98.7 % | 100.0 % |
| **Mapped reads** | 54.5 % | 93.4 % | 54.4 % | 93.9 % |
