# Supplementary material for: Snow alga Sanguina aurantia as revealed through de novo genome assembly and annotation
Source: G3 (Bethesda). 2024 Aug 2;14(10):jkae181. doi: 10.1093/g3journal/jkae181 (PMC11457085; doi:10.1093/g3journal/jkae181)
Supplement: jkae181_Supplementary_Data [file jkae181_supplementary_data.zip › Table_S4_G3-2024-405201.docx]

| **GO Class ID** | **Definitions** | **Counts**  **Genome A** | **Counts**  **Genome B** |
| --- | --- | --- | --- |
| [GO:0003674](http://amigo.geneontology.org/cgi-bin/amigo/go.cgi?action=query&view=details&search_constraint=terms&query=GO:0003674) | molecular_function | [512](javascript:ReverseDisplay('GO:0003674')) | [514](javascript:ReverseDisplay('GO:0003674')) |
| [GO:0008150](http://amigo.geneontology.org/cgi-bin/amigo/go.cgi?action=query&view=details&search_constraint=terms&query=GO:0008150) | biological_process | 380 | [384](javascript:ReverseDisplay('GO:0008150')) |
| [GO:0003824](http://amigo.geneontology.org/cgi-bin/amigo/go.cgi?action=query&view=details&search_constraint=terms&query=GO:0003824) | catalytic activity | 350 | [351](javascript:ReverseDisplay('GO:0003824')) |
| [GO:0009987](http://amigo.geneontology.org/cgi-bin/amigo/go.cgi?action=query&view=details&search_constraint=terms&query=GO:0009987) | cellular process | 294 | [296](javascript:ReverseDisplay('GO:0009987')) |
| [GO:0008152](http://amigo.geneontology.org/cgi-bin/amigo/go.cgi?action=query&view=details&search_constraint=terms&query=GO:0008152) | metabolic process | 259 | [264](javascript:ReverseDisplay('GO:0008152')) |
| [GO:0005575](http://amigo.geneontology.org/cgi-bin/amigo/go.cgi?action=query&view=details&search_constraint=terms&query=GO:0005575) | cellular_component | 133 | [135](javascript:ReverseDisplay('GO:0005575')) |
| [GO:0005623](http://amigo.geneontology.org/cgi-bin/amigo/go.cgi?action=query&view=details&search_constraint=terms&query=GO:0005623) | cell | 121 | [122](javascript:ReverseDisplay('GO:0005623')) |
| [GO:0009058](http://amigo.geneontology.org/cgi-bin/amigo/go.cgi?action=query&view=details&search_constraint=terms&query=GO:0009058) | biosynthetic process | 108 | [115](javascript:ReverseDisplay('GO:0009058')) |
| [GO:0005622](http://amigo.geneontology.org/cgi-bin/amigo/go.cgi?action=query&view=details&search_constraint=terms&query=GO:0005622) | intracellular | 109 | [112](javascript:ReverseDisplay('GO:0005622')) |
| [GO:0016740](http://amigo.geneontology.org/cgi-bin/amigo/go.cgi?action=query&view=details&search_constraint=terms&query=GO:0016740) | transferase activity | 117 | [109](javascript:ReverseDisplay('GO:0016740')) |
| [GO:0005488](http://amigo.geneontology.org/cgi-bin/amigo/go.cgi?action=query&view=details&search_constraint=terms&query=GO:0005488) | binding | 106 | [108](javascript:ReverseDisplay('GO:0005488')) |
| [GO:0006139](http://amigo.geneontology.org/cgi-bin/amigo/go.cgi?action=query&view=details&search_constraint=terms&query=GO:0006139) | nucleobase, nucleoside, nucleotide and nucleic acid metabolic process | 99 | [103](javascript:ReverseDisplay('GO:0006139')) |
| [GO:0016787](http://amigo.geneontology.org/cgi-bin/amigo/go.cgi?action=query&view=details&search_constraint=terms&query=GO:0016787) | hydrolase activity | 96 | [99](javascript:ReverseDisplay('GO:0016787')) |
| [GO:0019538](http://amigo.geneontology.org/cgi-bin/amigo/go.cgi?action=query&view=details&search_constraint=terms&query=GO:0019538) | protein metabolic process | [54](javascript:ReverseDisplay('GO:0019538')) | [55](javascript:ReverseDisplay('GO:0019538')) |
| [GO:0006810](http://amigo.geneontology.org/cgi-bin/amigo/go.cgi?action=query&view=details&search_constraint=terms&query=GO:0006810) | transport | 51 | [50](javascript:ReverseDisplay('GO:0006810')) |
| [GO:0016043](http://amigo.geneontology.org/cgi-bin/amigo/go.cgi?action=query&view=details&search_constraint=terms&query=GO:0016043) | cellular component organization and biogenesis | 51 | [50](javascript:ReverseDisplay('GO:0016043')) |
| [GO:0016020](http://amigo.geneontology.org/cgi-bin/amigo/go.cgi?action=query&view=details&search_constraint=terms&query=GO:0016020) | membrane | 41 | [44](javascript:ReverseDisplay('GO:0016020')) |
| [GO:0005737](http://amigo.geneontology.org/cgi-bin/amigo/go.cgi?action=query&view=details&search_constraint=terms&query=GO:0005737) | cytoplasm | 39 | [43](javascript:ReverseDisplay('GO:0005737')) |
| [GO:0005215](http://amigo.geneontology.org/cgi-bin/amigo/go.cgi?action=query&view=details&search_constraint=terms&query=GO:0005215) | transporter activity | 39 | [38](javascript:ReverseDisplay('GO:0005215')) |
| [GO:0005515](http://amigo.geneontology.org/cgi-bin/amigo/go.cgi?action=query&view=details&search_constraint=terms&query=GO:0005515) | protein binding | 28 | [26](javascript:ReverseDisplay('GO:0005515')) |
| [GO:0003676](http://amigo.geneontology.org/cgi-bin/amigo/go.cgi?action=query&view=details&search_constraint=terms&query=GO:0003676) | nucleic acid binding | 24 | [25](javascript:ReverseDisplay('GO:0003676')) |
| [GO:0006464](http://amigo.geneontology.org/cgi-bin/amigo/go.cgi?action=query&view=details&search_constraint=terms&query=GO:0006464) | protein modification process | 27 | [25](javascript:ReverseDisplay('GO:0006464')) |
| [GO:0005634](http://amigo.geneontology.org/cgi-bin/amigo/go.cgi?action=query&view=details&search_constraint=terms&query=GO:0005634) | nucleus | [22](javascript:ReverseDisplay('GO:0005634')) | [22](javascript:ReverseDisplay('GO:0005634')) |
| [GO:0009056](http://amigo.geneontology.org/cgi-bin/amigo/go.cgi?action=query&view=details&search_constraint=terms&query=GO:0009056) | catabolic process | 23 | [21](javascript:ReverseDisplay('GO:0009056')) |
| [GO:0006629](http://amigo.geneontology.org/cgi-bin/amigo/go.cgi?action=query&view=details&search_constraint=terms&query=GO:0006629) | lipid metabolic process | 22 | [19](javascript:ReverseDisplay('GO:0006629')) |
| [GO:0016301](http://amigo.geneontology.org/cgi-bin/amigo/go.cgi?action=query&view=details&search_constraint=terms&query=GO:0016301) | kinase activity | 22 | [19](javascript:ReverseDisplay('GO:0016301')) |
| [GO:0005975](http://amigo.geneontology.org/cgi-bin/amigo/go.cgi?action=query&view=details&search_constraint=terms&query=GO:0005975) | carbohydrate metabolic process | [18](javascript:ReverseDisplay('GO:0005975')) | [18](javascript:ReverseDisplay('GO:0005975')) |
| [GO:0004518](http://amigo.geneontology.org/cgi-bin/amigo/go.cgi?action=query&view=details&search_constraint=terms&query=GO:0004518) | nuclease activity | 15 | [18](javascript:ReverseDisplay('GO:0004518')) |
| [GO:0006259](http://amigo.geneontology.org/cgi-bin/amigo/go.cgi?action=query&view=details&search_constraint=terms&query=GO:0006259) | DNA metabolic process | 20 | [18](javascript:ReverseDisplay('GO:0006259')) |
| [GO:0006412](http://amigo.geneontology.org/cgi-bin/amigo/go.cgi?action=query&view=details&search_constraint=terms&query=GO:0006412) | translation | 13 | [16](javascript:ReverseDisplay('GO:0006412')) |
| [GO:0003723](http://amigo.geneontology.org/cgi-bin/amigo/go.cgi?action=query&view=details&search_constraint=terms&query=GO:0003723) | RNA binding | 16 | [15](javascript:ReverseDisplay('GO:0003723')) |
| [GO:0030234](http://amigo.geneontology.org/cgi-bin/amigo/go.cgi?action=query&view=details&search_constraint=terms&query=GO:0030234) | enzyme regulator activity | [12](javascript:ReverseDisplay('GO:0030234')) | [12](javascript:ReverseDisplay('GO:0030234')) |
| [GO:0006950](http://amigo.geneontology.org/cgi-bin/amigo/go.cgi?action=query&view=details&search_constraint=terms&query=GO:0006950) | response to stress | [12](javascript:ReverseDisplay('GO:0006950')) | [12](javascript:ReverseDisplay('GO:0006950')) |
| [GO:0000166](http://amigo.geneontology.org/cgi-bin/amigo/go.cgi?action=query&view=details&search_constraint=terms&query=GO:0000166) | nucleotide binding | 10 | [11](javascript:ReverseDisplay('GO:0000166')) |
| [GO:0005739](http://amigo.geneontology.org/cgi-bin/amigo/go.cgi?action=query&view=details&search_constraint=terms&query=GO:0005739) | mitochondrion | 9 | [11](javascript:ReverseDisplay('GO:0005739')) |
| [GO:0006091](http://amigo.geneontology.org/cgi-bin/amigo/go.cgi?action=query&view=details&search_constraint=terms&query=GO:0006091) | generation of precursor metabolites and energy | 8 | [10](javascript:ReverseDisplay('GO:0006091')) |
| [GO:0003677](http://amigo.geneontology.org/cgi-bin/amigo/go.cgi?action=query&view=details&search_constraint=terms&query=GO:0003677) | DNA binding | [7](javascript:ReverseDisplay('GO:0003677')) | [9](javascript:ReverseDisplay('GO:0003677')) |
| [GO:0005783](http://amigo.geneontology.org/cgi-bin/amigo/go.cgi?action=query&view=details&search_constraint=terms&query=GO:0005783) | endoplasmic reticulum | [7](javascript:ReverseDisplay('GO:0005783')) | [9](javascript:ReverseDisplay('GO:0005783')) |
| [GO:0007049](http://amigo.geneontology.org/cgi-bin/amigo/go.cgi?action=query&view=details&search_constraint=terms&query=GO:0007049) | cell cycle | [9](javascript:ReverseDisplay('GO:0007049')) | [9](javascript:ReverseDisplay('GO:0007049')) |
| [GO:0007154](http://amigo.geneontology.org/cgi-bin/amigo/go.cgi?action=query&view=details&search_constraint=terms&query=GO:0007154) | cell communication | [8](javascript:ReverseDisplay('GO:0007154')) | [8](javascript:ReverseDisplay('GO:0007154')) |
| [GO:0005856](http://amigo.geneontology.org/cgi-bin/amigo/go.cgi?action=query&view=details&search_constraint=terms&query=GO:0005856) | cytoskeleton | 10 | [8](javascript:ReverseDisplay('GO:0005856')) |
| [GO:0005654](http://amigo.geneontology.org/cgi-bin/amigo/go.cgi?action=query&view=details&search_constraint=terms&query=GO:0005654) | nucleoplasm | [7](javascript:ReverseDisplay('GO:0005654')) | [7](javascript:ReverseDisplay('GO:0005654')) |
| [GO:0007165](http://amigo.geneontology.org/cgi-bin/amigo/go.cgi?action=query&view=details&search_constraint=terms&query=GO:0007165) | signal transduction | [7](javascript:ReverseDisplay('GO:0007165')) | [7](javascript:ReverseDisplay('GO:0007165')) |
| [GO:0009579](http://amigo.geneontology.org/cgi-bin/amigo/go.cgi?action=query&view=details&search_constraint=terms&query=GO:0009579) | thylakoid | [6](javascript:ReverseDisplay('GO:0009579')) | [6](javascript:ReverseDisplay('GO:0009579')) |
| [GO:0005886](http://amigo.geneontology.org/cgi-bin/amigo/go.cgi?action=query&view=details&search_constraint=terms&query=GO:0005886) | plasma membrane | [6](javascript:ReverseDisplay('GO:0005886')) | [5](javascript:ReverseDisplay('GO:0005886')) |
| [GO:0030246](http://amigo.geneontology.org/cgi-bin/amigo/go.cgi?action=query&view=details&search_constraint=terms&query=GO:0030246) | carbohydrate binding | [4](javascript:ReverseDisplay('GO:0030246')) | [5](javascript:ReverseDisplay('GO:0030246')) |
| [GO:0015979](http://amigo.geneontology.org/cgi-bin/amigo/go.cgi?action=query&view=details&search_constraint=terms&query=GO:0015979) | photosynthesis | [4](javascript:ReverseDisplay('GO:0015979')) | [4](javascript:ReverseDisplay('GO:0015979')) |
| [GO:0005198](http://amigo.geneontology.org/cgi-bin/amigo/go.cgi?action=query&view=details&search_constraint=terms&query=GO:0005198) | structural molecule activity | [5](javascript:ReverseDisplay('GO:0005198')) | [4](javascript:ReverseDisplay('GO:0005198')) |
| [GO:0008289](http://amigo.geneontology.org/cgi-bin/amigo/go.cgi?action=query&view=details&search_constraint=terms&query=GO:0008289) | lipid binding | [4](javascript:ReverseDisplay('GO:0008289')) | [4](javascript:ReverseDisplay('GO:0008289')) |
| [GO:0005794](http://amigo.geneontology.org/cgi-bin/amigo/go.cgi?action=query&view=details&search_constraint=terms&query=GO:0005794) | Golgi apparatus | [4](javascript:ReverseDisplay('GO:0005794')) | [4](javascript:ReverseDisplay('GO:0005794')) |
| [GO:0003774](http://amigo.geneontology.org/cgi-bin/amigo/go.cgi?action=query&view=details&search_constraint=terms&query=GO:0003774) | motor activity | [3](javascript:ReverseDisplay('GO:0003774')) | [3](javascript:ReverseDisplay('GO:0003774')) |
| [GO:0019748](http://amigo.geneontology.org/cgi-bin/amigo/go.cgi?action=query&view=details&search_constraint=terms&query=GO:0019748) | secondary metabolic process | [3](javascript:ReverseDisplay('GO:0019748')) | [3](javascript:ReverseDisplay('GO:0019748')) |
| [GO:0005840](http://amigo.geneontology.org/cgi-bin/amigo/go.cgi?action=query&view=details&search_constraint=terms&query=GO:0005840) | ribosome | [2](javascript:ReverseDisplay('GO:0005840')) | [3](javascript:ReverseDisplay('GO:0005840')) |
| [GO:0008135](http://amigo.geneontology.org/cgi-bin/amigo/go.cgi?action=query&view=details&search_constraint=terms&query=GO:0008135) | translation factor activity, nucleic acid binding | [3](javascript:ReverseDisplay('GO:0008135')) | [3](javascript:ReverseDisplay('GO:0008135')) |
| [GO:0019725](http://amigo.geneontology.org/cgi-bin/amigo/go.cgi?action=query&view=details&search_constraint=terms&query=GO:0019725) | cell homeostasis | [2](javascript:ReverseDisplay('GO:0019725')) | [2](javascript:ReverseDisplay('GO:0019725')) |
| [GO:0005730](http://amigo.geneontology.org/cgi-bin/amigo/go.cgi?action=query&view=details&search_constraint=terms&query=GO:0005730) | nucleolus | [2](javascript:ReverseDisplay('GO:0005730')) | [2](javascript:ReverseDisplay('GO:0005730')) |
| [GO:0004872](http://amigo.geneontology.org/cgi-bin/amigo/go.cgi?action=query&view=details&search_constraint=terms&query=GO:0004872) | receptor activity | [2](javascript:ReverseDisplay('GO:0004872')) | [2](javascript:ReverseDisplay('GO:0004872')) |
| [GO:0005773](http://amigo.geneontology.org/cgi-bin/amigo/go.cgi?action=query&view=details&search_constraint=terms&query=GO:0005773) | vacuole | [1](javascript:ReverseDisplay('GO:0005773')) | [2](javascript:ReverseDisplay('GO:0005773')) |
| [GO:0003682](http://amigo.geneontology.org/cgi-bin/amigo/go.cgi?action=query&view=details&search_constraint=terms&query=GO:0003682) | chromatin binding | [2](javascript:ReverseDisplay('GO:0003682')) | [2](javascript:ReverseDisplay('GO:0003682')) |
| [GO:0009653](http://amigo.geneontology.org/cgi-bin/amigo/go.cgi?action=query&view=details&search_constraint=terms&query=GO:0009653) | anatomical structure morphogenesis | [0](javascript:ReverseDisplay('GO:0009653')) | [2](javascript:ReverseDisplay('GO:0009653')) |
| [GO:0005777](http://amigo.geneontology.org/cgi-bin/amigo/go.cgi?action=query&view=details&search_constraint=terms&query=GO:0005777) | peroxisome | [2](javascript:ReverseDisplay('GO:0005777')) | [2](javascript:ReverseDisplay('GO:0005777')) |
| [GO:0004871](http://amigo.geneontology.org/cgi-bin/amigo/go.cgi?action=query&view=details&search_constraint=terms&query=GO:0004871) | signal transducer activity | [1](javascript:ReverseDisplay('GO:0004871')) | [1](javascript:ReverseDisplay('GO:0004871')) |
| [GO:0009605](http://amigo.geneontology.org/cgi-bin/amigo/go.cgi?action=query&view=details&search_constraint=terms&query=GO:0009605) | response to external stimulus | [1](javascript:ReverseDisplay('GO:0009605')) | [1](javascript:ReverseDisplay('GO:0009605')) |
| [GO:0005576](http://amigo.geneontology.org/cgi-bin/amigo/go.cgi?action=query&view=details&search_constraint=terms&query=GO:0005576) | extracellular region | [0](javascript:ReverseDisplay('GO:0005576')) | [1](javascript:ReverseDisplay('GO:0005576')) |
| [GO:0003700](http://amigo.geneontology.org/cgi-bin/amigo/go.cgi?action=query&view=details&search_constraint=terms&query=GO:0003700) | transcription factor activity | [1](javascript:ReverseDisplay('GO:0003700')) | [1](javascript:ReverseDisplay('GO:0003700')) |
| [GO:0009991](http://amigo.geneontology.org/cgi-bin/amigo/go.cgi?action=query&view=details&search_constraint=terms&query=GO:0009991) | response to extracellular stimulus | [1](javascript:ReverseDisplay('GO:0009991')) | [1](javascript:ReverseDisplay('GO:0009991')) |
| [GO:0030154](http://amigo.geneontology.org/cgi-bin/amigo/go.cgi?action=query&view=details&search_constraint=terms&query=GO:0030154) | cell differentiation | [0](javascript:ReverseDisplay('GO:0030154')) | [1](javascript:ReverseDisplay('GO:0030154')) |
| [GO:0005768](http://amigo.geneontology.org/cgi-bin/amigo/go.cgi?action=query&view=details&search_constraint=terms&query=GO:0005768) | endosome | [0](javascript:ReverseDisplay('GO:0005768')) | [1](javascript:ReverseDisplay('GO:0005768')) |
| [GO:0007275](http://amigo.geneontology.org/cgi-bin/amigo/go.cgi?action=query&view=details&search_constraint=terms&query=GO:0007275) | multicellular organismal development | [1](javascript:ReverseDisplay('GO:0007275')) | [1](javascript:ReverseDisplay('GO:0007275')) |
| [GO:0005635](http://amigo.geneontology.org/cgi-bin/amigo/go.cgi?action=query&view=details&search_constraint=terms&query=GO:0005635) | nuclear envelope | [1](javascript:ReverseDisplay('GO:0005635')) | [1](javascript:ReverseDisplay('GO:0005635')) |
