# Supplementary material for: Snow alga Sanguina aurantia as revealed through de novo genome assembly and annotation
Source: G3 (Bethesda). 2024 Aug 2;14(10):jkae181. doi: 10.1093/g3journal/jkae181 (PMC11457085; doi:10.1093/g3journal/jkae181)
Supplement: jkae181_Supplementary_Data [file jkae181_supplementary_data.zip › Table_S5_G3-2024-405201.docx]

| **category1** | **category2** | **ko_id** | **species_name** | **hsds_id** | **hsds_num** |
| --- | --- | --- | --- | --- | --- |
| 09101 Carbohydrate metabolism | 00010 Glycolysis / Gluconeogenesis [PATH:ko00010] | K01568  PC, pdc; pyruvate decarboxylase | S. aurnatia Genome A | g9994.t1 | 1 |
| 09101 Carbohydrate metabolism | 00053 Ascorbate and aldarate metabolism [PATH:ko00053] | K10046  GME; GP--mannose 3', 5'-epimerase | S. aurnatia Genome A | g2185.t1 | 1 |
| 09101 Carbohydrate metabolism | 00053 Ascorbate and aldarate metabolism [PATH:ko00053] | K10047  VTC4; inositol-phosphate phosphatase / L-galactose 1-phosphate phosphatase | S. aurnatia Genome A | g476.t1 | 1 |
| 09101 Carbohydrate metabolism | 00053 Ascorbate and aldarate metabolism [PATH:ko00053] | K00225  GLH; L-galactono-1,4-lactone dehydrogenase | S. aurnatia Genome A | g3548.t1 | 1 |
| 09103 Lipid metabolism | 00071 Fatty acid degradation [PATH:ko00071] | K10527  MFP2; enoyl-CoA hydratase/3-hydroxyacyl-CoA dehydrogenase | S. aurnatia Genome A | g4858.t1 | 1 |
| 09103 Lipid metabolism | 00100 Steroid biosynthesis [PATH:ko00100] | K07748  NSHL, ERG26; sterol-4alpha-carboxylate 3-dehydrogenase (decarboxylating) | S. aurnatia Genome A | g6313.t1 | 1 |
| 09102 Energy metabolism | 00190 Oxidative phosphorylation [PATH:ko00190] | K02113  ATPF1, atpH; F-type H+-transporting ATPase subunit delta | S. aurnatia Genome A | g3606.t1 | 1 |
| 09102 Energy metabolism | 00190 Oxidative phosphorylation [PATH:ko00190] | K02151  ATPeV1F, ATP6S14; V-type H+-transporting ATPase subunit F | S. aurnatia Genome A | g4590.t1 | 1 |
| 09102 Energy metabolism | 00195 Photosynthesis [PATH:ko00195] | K02695  psaH; photosystem I subunit VI | S. aurnatia Genome A | g8528.t1 | 1 |
| 09102 Energy metabolism | 00196 Photosynthesis - antenna proteins [PATH:ko00196] | K08912  LHCB1; light-harvesting complex II chlorophyll a/b binding protein 1 | S. aurnatia Genome A | g2842.t1, g9814.t1 | 2 |
| 09102 Energy metabolism | 00196 Photosynthesis - antenna proteins [PATH:ko00196] | K08913  LHCB2; light-harvesting complex II chlorophyll a/b binding protein 2 | S. aurnatia Genome A | g4826.t1, g9814.t1 | 2 |
| 09104 Nucleotide metabolism | 00230 Purine metabolism [PATH:ko00230] | K11808  AE2; phosphoribosylaminoimidazole carboxylase | S. aurnatia Genome A | g3438.t1 | 1 |
| 09104 Nucleotide metabolism | 00230 Purine metabolism [PATH:ko00230] | K10808  RRM2; ribonucleoside-diphosphate reductase subunit M2 | S. aurnatia Genome A | g571.t2 | 1 |
| 09103 Lipid metabolism | 00561 Glycerolipid metabolism [PATH:ko00561] | K03715  MG; 1,2-diacylglycerol 3-beta-galactosyltransferase | S. aurnatia Genome A | g7934.t1 | 1 |
| 09101 Carbohydrate metabolism | 00562 Inositol phosphate metabolism [PATH:ko00562] | K15422  SAL; 3'(2'), 5'-bisphosphate nucleotidase / inositol polyphosphate 1-phosphatase | S. aurnatia Genome A | g1738.t1 | 1 |
| 09103 Lipid metabolism | 00590 Arachidonic acid metabolism [PATH:ko00590] | K00079  CBR1; carbonyl reductase 1 | S. aurnatia Genome A | g4183.t1 | 1 |
| 09101 Carbohydrate metabolism | 00630 Glyoxylate and dicarboxylate metabolism [PATH:ko00630] | K01602  rbcS, cbbS; ribulose-bisphosphate carboxylase small chain | S. aurnatia Genome A | g4001.t1 | 1 |
| 09102 Energy metabolism | 00680 Methane metabolism [PATH:ko00680] | K00831  serC, PSAT1; phosphoserine aminotransferase | S. aurnatia Genome A | g8090.t1 | 1 |
| 09108 Metabolism of cofactors and vitamins | 00730 Thiamine metabolism [PATH:ko00730] | K03146  THI4, THI1; cysteine-dependent adenosine diphosphate thiazole synthase | S. aurnatia Genome A | g5124.t1 | 1 |
| 09108 Metabolism of cofactors and vitamins | 00760 Nicotinate and nicotinamide metabolism [PATH:ko00760] | K03426  E3.6.1.22, NUT12, nudC; NA+ diphosphatase | S. aurnatia Genome A | g2934.t1 | 1 |
| 09108 Metabolism of cofactors and vitamins | 00860 Porphyrin and chlorophyll metabolism [PATH:ko00860] | K10960  chlP, bchP; geranylgeranyl diphosphate/geranylgeranyl-bacteriochlorophyllide a reductase | S. aurnatia Genome A | g5014.t1 | 1 |
| 09122 Translation | 03010 Ribosome [PATH:ko03010] | K02879  RP-L17, MRPL17, rplQ; large subunit ribosomal protein L17 | S. aurnatia Genome A | g5820.t1 | 1 |
| 09122 Translation | 03010 Ribosome [PATH:ko03010] | K02909  RP-L31, rpmE; large subunit ribosomal protein L31 | S. aurnatia Genome A | g8337.t1 | 1 |
| 09122 Translation | 03010 Ribosome [PATH:ko03010] | K02940  RP-L9e, RPL9; large subunit ribosomal protein L9e | S. aurnatia Genome A | g4416.t1 | 1 |
| 09182 Protein families: genetic information processing | 03012 Translation factors [BR:ko03012] | K02835  prfA, MTRF1, MRF1; peptide chain release factor 1 | S. aurnatia Genome A | g8227.t1 | 1 |
| 09122 Translation | 03015 mRNA surveillance pathway [PATH:ko03015] | K03267  ERF3, GSPT; peptide chain release factor subunit 3 | S. aurnatia Genome A | g6115.t1 | 1 |
| 09182 Protein families: genetic information processing | 03021 Transcription machinery [BR:ko03021] | K24104  GPN; GPN-loop GTPase | S. aurnatia Genome A | g1279.t1 | 1 |
| 09121 Transcription | 03022 Basal transcription factors [PATH:ko03022] | K10843  ERCC3, XPB; NA excision repair protein ERCC-3 | S. aurnatia Genome A | g7902.t1 | 1 |
| 09182 Protein families: genetic information processing | 03029 Mitochondrial biogenesis [BR:ko03029] | K22066  BOLA1; BolA-like protein 1 | S. aurnatia Genome A | g833.t1 | 1 |
| 09124 Replication and repair | 03030 DNA replication [PATH:ko03030] | K02212  MCM4, CC54; NA replication licensing factor MCM4 | S. aurnatia Genome A | g8811.t1 | 1 |
| 09182 Protein families: genetic information processing | 03036 hromosome and associated proteins [BR:ko03036] | K11279  NAP1L1, NRP; nucleosome assembly protein 1-like 1 | S. aurnatia Genome A | g3000.t1 | 1 |
| 09182 Protein families: genetic information processing | 03036 hromosome and associated proteins [BR:ko03036] | K22767  MCC1; histone acetyltransferase MCC1 | S. aurnatia Genome A | g7863.t1 | 1 |
| 09121 Transcription | 03040 Spliceosome [PATH:ko03040] | K12823  X5, BP2; ATP-dependent RNA helicase X5/BP2 | S. aurnatia Genome A | g4466.t1 | 1 |
| 09121 Transcription | 03040 Spliceosome [PATH:ko03040] | K12867  SYF1, XAB2; pre-mRNA-splicing factor SYF1 | S. aurnatia Genome A | g6417.t1 | 1 |
| 09182 Protein families: genetic information processing | 03041 Spliceosome [BR:ko03041] | K13125  NOSIP; nitric oxide synthase-interacting protein | S. aurnatia Genome A | g6027.t1 | 1 |
| 09123 Folding, sorting and degradation | 03050 Proteasome [PATH:ko03050] | K03039  PSM13, RPN9; 26S proteasome regulatory subunit N9 | S. aurnatia Genome A | g5767.t1 | 1 |
| 09123 Folding, sorting and degradation | 03050 Proteasome [PATH:ko03050] | K02732  PSMB1; 20S proteasome subunit beta 6 | S. aurnatia Genome A | g5824.t1 | 1 |
| 09132 Signal transduction | 04068 FoxO signaling pathway [PATH:ko04068] | K11434  PRMT1; type I protein arginine methyltransferase | S. aurnatia Genome A | g6016.t1 | 1 |
| 09182 Protein families: genetic information processing | 04121 Ubiquitin system [BR:ko04121] | K10638  UHRF1, NP95; E3 ubiquitin-protein ligase UHRF1 | S. aurnatia Genome A | g5340.t1 | 1 |
| 09182 Protein families: genetic information processing | 04131 Membrane trafficking [BR:ko04131] | K20289  COG2; conserved oligomeric Golgi complex subunit 2 | S. aurnatia Genome A | g2791.t1 | 1 |
| 09182 Protein families: genetic information processing | 04131 Membrane trafficking [BR:ko04131] | K22940  YIPF1_2; protein YIPF1/2 | S. aurnatia Genome A | g5959.t1 | 1 |
| 09182 Protein families: genetic information processing | 04131 Membrane trafficking [BR:ko04131] | K20523  SH3YL1; SH3 domain-containing YSC84-like protein 1 | S. aurnatia Genome A | g4449.t1 | 1 |
| 09123 Folding, sorting and degradation | 04141 Protein processing in endoplasmic reticulum [PATH:ko04141] | K11718  HUGT; UP-glucose:glycoprotein glucosyltransferase | S. aurnatia Genome A | g8963.t1 | 1 |
| 09141 Transport and catabolism | 04145 Phagosome [PATH:ko04145] | K10414  YNC2H, NCH2; dynein heavy chain 2, cytosolic | S. aurnatia Genome A | g5791.t1 | 1 |
| 09141 Transport and catabolism | 04145 Phagosome [PATH:ko04145] | K07375  TUBB; tubulin beta | S. aurnatia Genome A | g723.t1 | 1 |
| 09141 Transport and catabolism | 04146 Peroxisome [PATH:ko04146] | K13237  ECR2, SPS19; 2,4-dienoyl-CoA reductase [(3E)-enoyl-CoA-producing], peroxisomal | S. aurnatia Genome A | g4069.t1 | 1 |
| 09143 Cell growth and death | 04217 Necroptosis [PATH:ko04217] | K11251  H2A; histone H2A | S. aurnatia Genome A | g827.t1 | 1 |
| 09164 Neurodegenerative disease | 05014 Amyotrophic lateral sclerosis [PATH:ko05014] | K11518  TOM40; mitochondrial import receptor subunit TOM40 | S. aurnatia Genome A | g10136.t1 | 1 |
| 09161 Cancer: overview | 05202 Transcriptional misregulation in cancer [PATH:ko05202] | K11253  H3; histone H3 | S. aurnatia Genome A | g2632.t1 | 1 |
| 09161 Cancer: overview | 05203 Viral carcinogenesis [PATH:ko05203] | K11252  H2B; histone H2B | S. aurnatia Genome A | g826.t1 | 1 |
| 09161 Cancer: overview | 05203 Viral carcinogenesis [PATH:ko05203] | K11254  H4; histone H4 | S. aurnatia Genome A | g4288.t1 | 1 |
| 09191 Unclassified: metabolism | 99980 Enzymes with E numbers | K23856  GPX; peroxiredoxin | S. aurnatia Genome A | g5463.t1 | 1 |
| 09191 Unclassified: metabolism | 99980 Enzymes with E numbers | K11206  NIT1, ybeM; deaminated glutathione amidase | S. aurnatia Genome A | g6957.t1 | 1 |
| 09191 Unclassified: metabolism | 99987 ofactor metabolism | K18586  COQ4; ubiquinone biosynthesis protein COQ4 | S. aurnatia Genome A | g5860.t1 | 1 |
| 09194 Poorly characterized | 99997 Function unknown | K07034  K07034; uncharacterized protein | S. aurnatia Genome A | g1295.t1 | 1 |
| 09101 Carbohydrate metabolism | 00010 Glycolysis / Gluconeogenesis [PATH:ko00010] | K00627  LAT, aceF, pdhC; pyruvate dehydrogenase E2 component (dihydrolipoamide acetyltransferase) | S. aurantia Genome B | g1737.t1 | 1 |
| 09101 Carbohydrate metabolism | 00020 itrate cycle (TA cycle) [PATH:ko00020] | K01900  LSC2; succinyl-CoA synthetase beta subunit | S. aurantia Genome B | g6591.t1 | 1 |
| 09101 Carbohydrate metabolism | 00052 Galactose metabolism [PATH:ko00052] | K01854  glf; UP-galactopyranose mutase | S. aurantia Genome B | g1735.t1 | 1 |
| 09103 Lipid metabolism | 00100 Steroid biosynthesis [PATH:ko00100] | K05917  CYP51; sterol 14alpha-demethylase | S. aurantia Genome B | g6205.t1 | 1 |
| 09103 Lipid metabolism | 00100 Steroid biosynthesis [PATH:ko00100] | K07748  NSHL, ERG26; sterol-4alpha-carboxylate 3-dehydrogenase (decarboxylating) | S. aurantia Genome B | g8670.t1 | 1 |
| 09108 Metabolism of cofactors and vitamins | 00130 Ubiquinone and other terpenoid-quinone biosynthesis [PATH:ko00130] | K23095  MENG, menG; demethylphylloquinol methyltransferase | S. aurantia Genome B | g11213.t1 | 1 |
| 09103 Lipid metabolism | 00140 Steroid hormone biosynthesis [PATH:ko00140] | K12345  SR5A3; 3-oxo-5-alpha-steroid 4-dehydrogenase 3 / polyprenol reductase | S. aurantia Genome B | g6529.t1 | 1 |
| 09102 Energy metabolism | 00190 Oxidative phosphorylation [PATH:ko00190] | K03936  NUFS3; NAH dehydrogenase (ubiquinone) Fe-S protein 3 | S. aurantia Genome B | g8917.t1 | 1 |
| 09102 Energy metabolism | 00190 Oxidative phosphorylation [PATH:ko00190] | K02151  ATPeV1F, ATP6S14; V-type H+-transporting ATPase subunit F | S. aurantia Genome B | g11626.t1 | 1 |
| 09102 Energy metabolism | 00196 Photosynthesis - antenna proteins [PATH:ko00196] | K08912  LHCB1; light-harvesting complex II chlorophyll a/b binding protein 1 | S. aurantia Genome B | g5011.t1 | 1 |
| 09105 Amino acid metabolism | 00220 Arginine biosynthesis [PATH:ko00220] | K01941  E6.3.4.6; urea carboxylase | S. aurantia Genome B | g9955.t1 | 1 |
| 09104 Nucleotide metabolism | 00230 Purine metabolism [PATH:ko00230] | K10808  RRM2; ribonucleoside-diphosphate reductase subunit M2 | S. aurantia Genome B | g738.t1 | 1 |
| 09105 Amino acid metabolism | 00260 Glycine, serine and threonine metabolism [PATH:ko00260] | K01696  trpB; tryptophan synthase beta chain | S. aurantia Genome B | g4241.t1 | 1 |
| 09105 Amino acid metabolism | 00270 ysteine and methionine metabolism [PATH:ko00270] | K00558  NMT1, dcm; NA (cytosine-5)-methyltransferase 1 | S. aurantia Genome B | g3148.t1 | 1 |
| 09105 Amino acid metabolism | 00270 ysteine and methionine metabolism [PATH:ko00270] | K01251  E3.3.1.1, ahcY; adenosylhomocysteinase | S. aurantia Genome B | g2293.t1 | 1 |
| 09105 Amino acid metabolism | 00280 Valine, leucine and isoleucine degradation [PATH:ko00280] | K00253  IV, ivd; isovaleryl-CoA dehydrogenase | S. aurantia Genome B | g2461.t1 | 1 |
| 09105 Amino acid metabolism | 00280 Valine, leucine and isoleucine degradation [PATH:ko00280] | K01969  E6.4.1.4B; 3-methylcrotonyl-CoA carboxylase beta subunit | S. aurantia Genome B | g7001.t1 | 1 |
| 09101 Carbohydrate metabolism | 00500 Starch and sucrose metabolism [PATH:ko00500] | K00700  GBE1, glgB; 1,4-alpha-glucan branching enzyme | S. aurantia Genome B | g9216.t1 | 1 |
| 09103 Lipid metabolism | 00561 Glycerolipid metabolism [PATH:ko00561] | K00630  ATS1; glycerol-3-phosphate O-acyltransferase | S. aurantia Genome B | g4783.t1 | 1 |
| 09103 Lipid metabolism | 00561 Glycerolipid metabolism [PATH:ko00561] | K03715  MG; 1,2-diacylglycerol 3-beta-galactosyltransferase | S. aurantia Genome B | g9353.t1 | 1 |
| 09103 Lipid metabolism | 00564 Glycerophospholipid metabolism [PATH:ko00564] | K14286  AGXT2L1, ETNPPL; ethanolamine-phosphate phospho-lyase | S. aurantia Genome B | g10562.t1 | 1 |
| 09101 Carbohydrate metabolism | 00620 Pyruvate metabolism [PATH:ko00620] | K00626  ACAT, atoB; acetyl-CoA C-acetyltransferase | S. aurantia Genome B | g1434.t1 | 1 |
| 09101 Carbohydrate metabolism | 00630 Glyoxylate and dicarboxylate metabolism [PATH:ko00630] | K01602  rbcS, cbbS; ribulose-bisphosphate carboxylase small chain | S. aurantia Genome B | g864.t1 | 1 |
| 09108 Metabolism of cofactors and vitamins | 00730 Thiamine metabolism [PATH:ko00730] | K03146  THI4, THI1; cysteine-dependent adenosine diphosphate thiazole synthase | S. aurantia Genome B | g4720.t1 | 1 |
| 09108 Metabolism of cofactors and vitamins | 00780 Biotin metabolism [PATH:ko00780] | K00652  bioF; 8-amino-7-oxononanoate synthase | S. aurantia Genome B | g9094.t1 | 1 |
| 09108 Metabolism of cofactors and vitamins | 00860 Porphyrin and chlorophyll metabolism [PATH:ko00860] | K21480  HO, pbsA1, hmuO; heme oxygenase (biliverdin-producing, ferredoxin) | S. aurantia Genome B | g5165.t1 | 1 |
| 09102 Energy metabolism | 00920 Sulfur metabolism [PATH:ko00920] | K01738  cysK; cysteine synthase | S. aurantia Genome B | g3920.t1 | 1 |
| 09122 Translation | 00970 Aminoacyl-tRNA biosynthesis [PATH:ko00970] | K01881  PARS, proS; prolyl-tRNA synthetase | S. aurantia Genome B | g1629.t1 | 1 |
| 09181 Protein families: metabolism | 01001 Protein kinases [BR:ko01001] | K08869  ACK, ABC1; aarF domain-containing kinase | S. aurantia Genome B | g2125.t1 | 1 |
| 09183 Protein families: signaling and cellular processes | 02000 Transporters [BR:ko02000] | K15285  SLC35E3; solute carrier family 35, member E3 | S. aurantia Genome B | g1842.t1 | 1 |
| 09131 Membrane transport | 02010 AB transporters [PATH:ko02010] | K05681  ABCG2, C338; ATP-binding cassette, subfamily G (WHITE), member 2 | S. aurantia Genome B | g2712.t1 | 1 |
| 09122 Translation | 03010 Ribosome [PATH:ko03010] | K02998  RP-SAe, RPSA; small subunit ribosomal protein SAe | S. aurantia Genome B | g696.t1 | 1 |
| 09122 Translation | 03010 Ribosome [PATH:ko03010] | K02871  RP-L13, MRPL13, rplM; large subunit ribosomal protein L13 | S. aurantia Genome B | g10184.t1 | 1 |
| 09122 Translation | 03010 Ribosome [PATH:ko03010] | K02940  RP-L9e, RPL9; large subunit ribosomal protein L9e | S. aurantia Genome B | g403.t1 | 1 |
| 09122 Translation | 03010 Ribosome [PATH:ko03010] | K02927  RP-L40e, RPL40, UBA52; ubiquitin-large subunit ribosomal protein L40e | S. aurantia Genome B | g847.t1 | 1 |
| 09122 Translation | 03013 RNA transport [PATH:ko03013] | K03247  EIF3H; translation initiation factor 3 subunit H | S. aurantia Genome B | g5333.t1 | 1 |
| 09123 Folding, sorting and degradation | 03018 RNA degradation [PATH:ko03018] | K12598  MTR4, SKIV2L2; ATP-dependent RNA helicase OB1 | S. aurantia Genome B | g9871.t1 | 1 |
| 09121 Transcription | 03020 RNA polymerase [PATH:ko03020] | K14721  RPC5, POLR3E; NA-directed RNA polymerase III subunit RPC5 | S. aurantia Genome B | g7061.t2 | 1 |
| 09182 Protein families: genetic information processing | 03021 Transcription machinery [BR:ko03021] | K24104  GPN; GPN-loop GTPase | S. aurantia Genome B | g97.t1 | 1 |
| 09182 Protein families: genetic information processing | 03029 Mitochondrial biogenesis [BR:ko03029] | K17679  MSS116; ATP-dependent RNA helicase MSS116, mitochondrial | S. aurantia Genome B | g1236.t1 | 1 |
| 09182 Protein families: genetic information processing | 03029 Mitochondrial biogenesis [BR:ko03029] | K22066  BOLA1; BolA-like protein 1 | S. aurantia Genome B | g11032.t1 | 1 |
| 09124 Replication and repair | 03030 DNA replication [PATH:ko03030] | K02212  MCM4, CC54; NA replication licensing factor MCM4 | S. aurantia Genome B | g2286.t1 | 1 |
| 09121 Transcription | 03040 Spliceosome [PATH:ko03040] | K12837  U2AF2; splicing factor U2AF 65 ka subunit | S. aurantia Genome B | g11340.t1 | 1 |
| 09121 Transcription | 03040 Spliceosome [PATH:ko03040] | K03283  HSPA1s; heat shock 70ka protein 1/2/6/8 | S. aurantia Genome B | g11368.t1 | 1 |
| 09121 Transcription | 03040 Spliceosome [PATH:ko03040] | K12867  SYF1, XAB2; pre-mRNA-splicing factor SYF1 | S. aurantia Genome B | g8540.t1 | 1 |
| 09121 Transcription | 03040 Spliceosome [PATH:ko03040] | K12873  BU31, G10; bud site selection protein 31 | S. aurantia Genome B | g6962.t1 | 1 |
| 09182 Protein families: genetic information processing | 03041 Spliceosome [BR:ko03041] | K13106  BU13, CWC26; pre-mRNA-splicing factor CWC26 | S. aurantia Genome B | g4608.t1 | 1 |
| 09182 Protein families: genetic information processing | 03041 Spliceosome [BR:ko03041] | K13125  NOSIP; nitric oxide synthase-interacting protein | S. aurantia Genome B | g9299.t1 | 1 |
| 09123 Folding, sorting and degradation | 03050 Proteasome [PATH:ko03050] | K03039  PSM13, RPN9; 26S proteasome regulatory subunit N9 | S. aurantia Genome B | g9040.t1 | 1 |
| 09132 Signal transduction | 04016 MAPK signaling pathway - plant [PATH:ko04016] | K14498  SNRK2; serine/threonine-protein kinase SRK2 | S. aurantia Genome B | g9362.t1 | 1 |
| 09132 Signal transduction | 04068 FoxO signaling pathway [PATH:ko04068] | K11434  PRMT1; type I protein arginine methyltransferase | S. aurantia Genome B | g9285.t1 | 1 |
| 09123 Folding, sorting and degradation | 04120 Ubiquitin mediated proteolysis [PATH:ko04120] | K03355  APC8, CC23; anaphase-promoting complex subunit 8 | S. aurantia Genome B | g3267.t1 | 1 |
| 09182 Protein families: genetic information processing | 04121 Ubiquitin system [BR:ko04121] | K10638  UHRF1, NP95; E3 ubiquitin-protein ligase UHRF1 | S. aurantia Genome B | g6240.t1 | 1 |
| 09182 Protein families: genetic information processing | 04131 Membrane trafficking [BR:ko04131] | K20289  COG2; conserved oligomeric Golgi complex subunit 2 | S. aurantia Genome B | g4952.t1 | 1 |
| 09141 Transport and catabolism | 04138 Autophagy - yeast [PATH:ko04138] | K20179  VPS11, PEP5; vacuolar protein sorting-associated protein 11 | S. aurantia Genome B | g1936.t1 | 1 |
| 09141 Transport and catabolism | 04142 Lysosome [PATH:ko04142] | K08568  CTSZ; cathepsin X | S. aurantia Genome B | g8757.t1 | 1 |
| 09141 Transport and catabolism | 04145 Phagosome [PATH:ko04145] | K07375  TUBB; tubulin beta | S. aurantia Genome B | g3754.t1 | 1 |
| 09141 Transport and catabolism | 04146 Peroxisome [PATH:ko04146] | K13237  ECR2, SPS19; 2,4-dienoyl-CoA reductase [(3E)-enoyl-CoA-producing], peroxisomal | S. aurantia Genome B | g790.t1 | 1 |
| 09143 Cell growth and death | 04214 Apoptosis - fly [PATH:ko04214] | K03386  PRX2_4, ahpC; peroxiredoxin 2/4 | S. aurantia Genome B | g11503.t1 | 1 |
| 09143 Cell growth and death | 04217 Necroptosis [PATH:ko04217] | K11251  H2A; histone H2A | S. aurantia Genome B | g493.t1 | 1 |
| 09159 Environmental adaptation | 04714 Thermogenesis [PATH:ko04714] | K18163  NUFAF6; NAH dehydrogenase [ubiquinone] 1 alpha subcomplex assembly factor 6 | S. aurantia Genome B | g4156.t1 | 1 |
| 09183 Protein families: signaling and cellular processes | 04812 Cytoskeleton proteins [BR:ko04812] | K21766  TBCC; tubulin-specific chaperone C | S. aurantia Genome B | g2273.t1 | 1 |
| 09161 Cancer: overview | 05203 Viral carcinogenesis [PATH:ko05203] | K11252  H2B; histone H2B | S. aurantia Genome B | g494.t1 | 1 |
| 09161 Cancer: overview | 05203 Viral carcinogenesis [PATH:ko05203] | K11254  H4; histone H4 | S. aurantia Genome B | g495.t1 | 1 |
| 09191 Unclassified: metabolism | 99980 Enzymes with E numbers | K11206  NIT1, ybeM; deaminated glutathione amidase | S. aurantia Genome B | g6466.t1 | 1 |
| 09191 Unclassified: metabolism | 99980 Enzymes with E numbers | K01974  RTCA, rtcA; RNA 3'-terminal phosphate cyclase (ATP) | S. aurantia Genome B | g8300.t1 | 1 |
| 09191 Unclassified: metabolism | 99987 ofactor metabolism | K18586  COQ4; ubiquinone biosynthesis protein COQ4 | S. aurantia Genome B | g9136.t1 | 1 |
| 09194 Poorly characterized | 99996 General function prediction only | K03975  dedA; membrane-associated protein | S. aurantia Genome B | g231.t1 | 1 |
| 09194 Poorly characterized | 99997 Function unknown | K07034  K07034; uncharacterized protein | S. aurantia Genome B | g77.t1, g76.t1 | 2 |
