# Supplementary figures and images for: Snow alga Sanguina aurantia as revealed through de novo genome assembly and annotation
Source: G3 (Bethesda). 2024 Aug 2;14(10):jkae181. doi: 10.1093/g3journal/jkae181 (PMC11457085; doi:10.1093/g3journal/jkae181)

## GenomeScope Profile

len:201,827,566bp uniq:49.9% het:0.828% kcov:18.3 err:0.559% dup:0.808% k:21

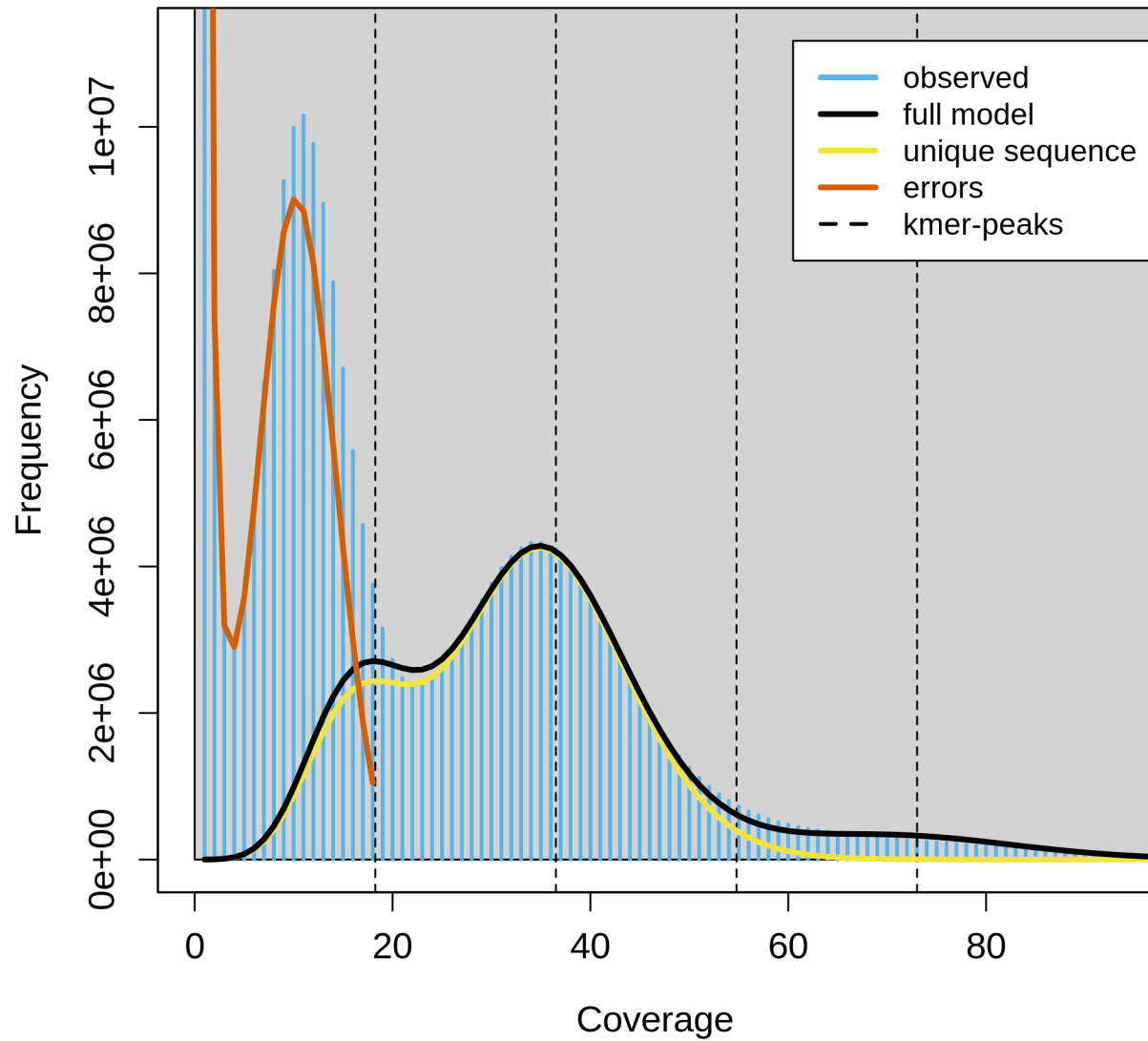

Supplement: jkae181_Supplementary_Data [file jkae181_supplementary_data.zip › Figure_S2_G3-2024-405201.pdf]

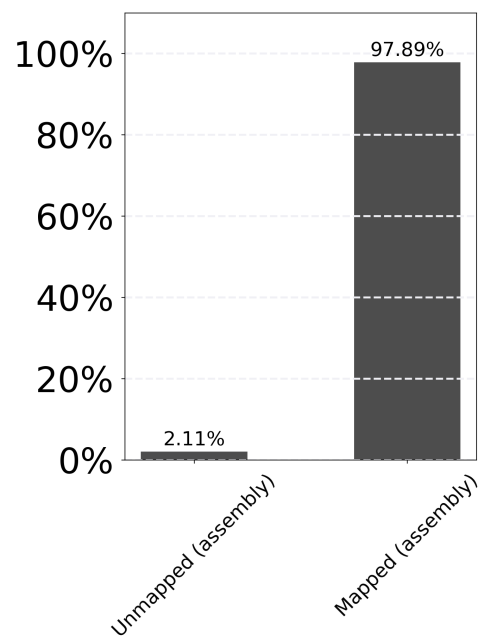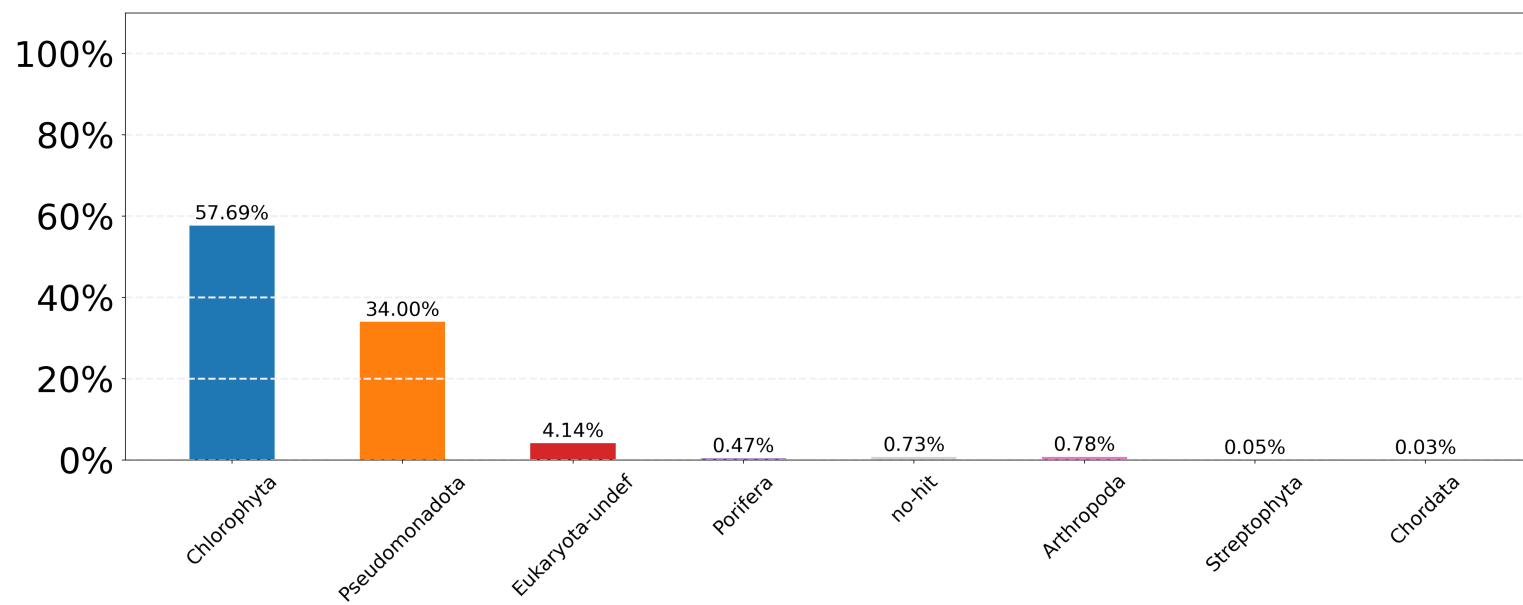

Supplement: jkae181_Supplementary_Data [file jkae181_supplementary_data.zip › Figure_S3_G3-2024-405201.pdf]

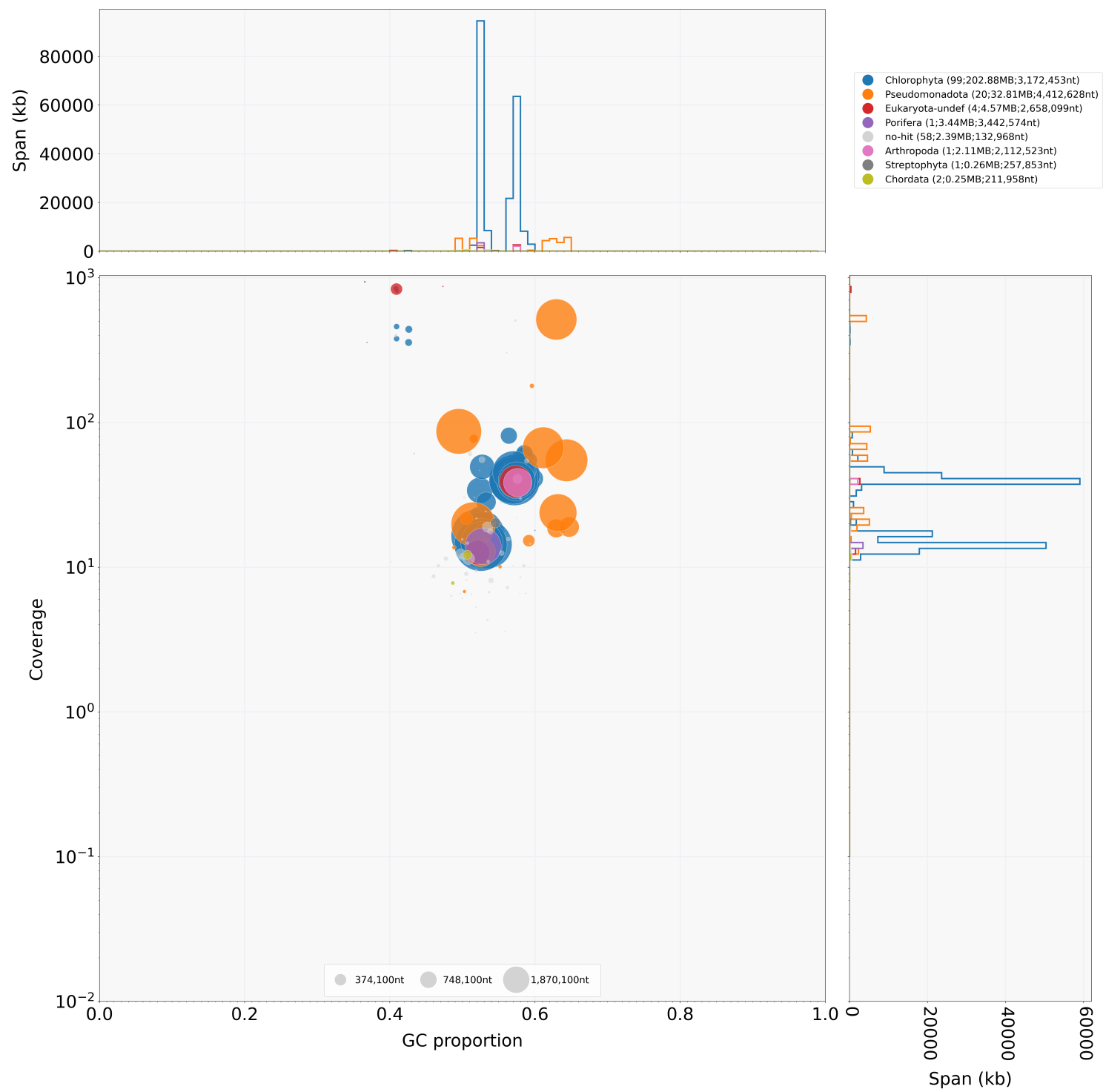

Supplement: jkae181_Supplementary_Data [file jkae181_supplementary_data.zip › Figure_S4_G3-2024-405201.pdf]

**a**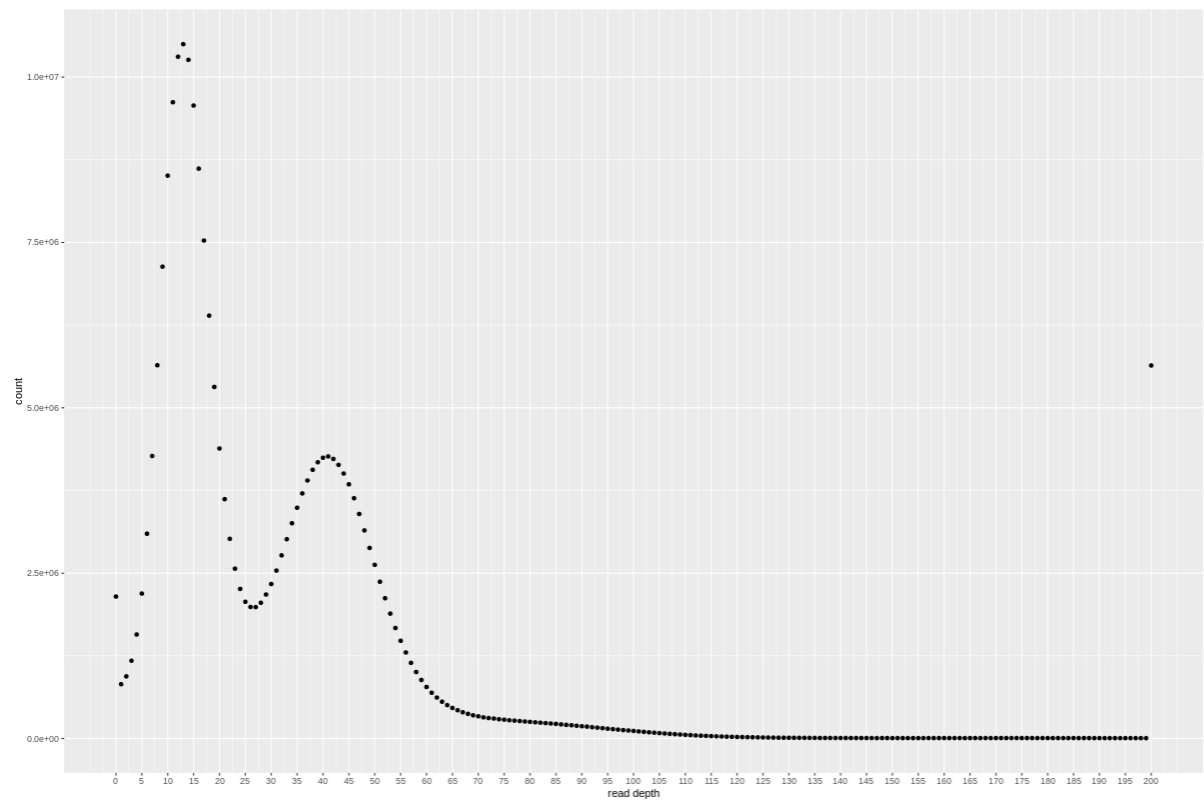**b**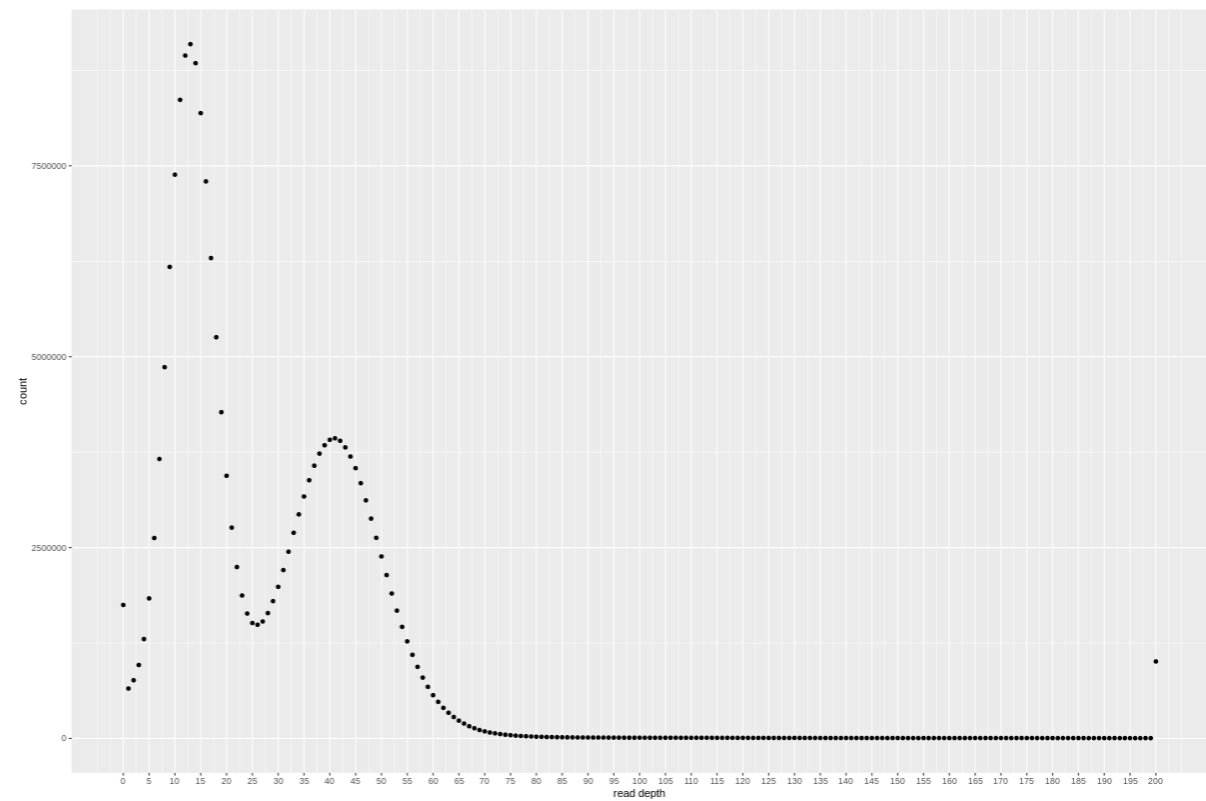**c**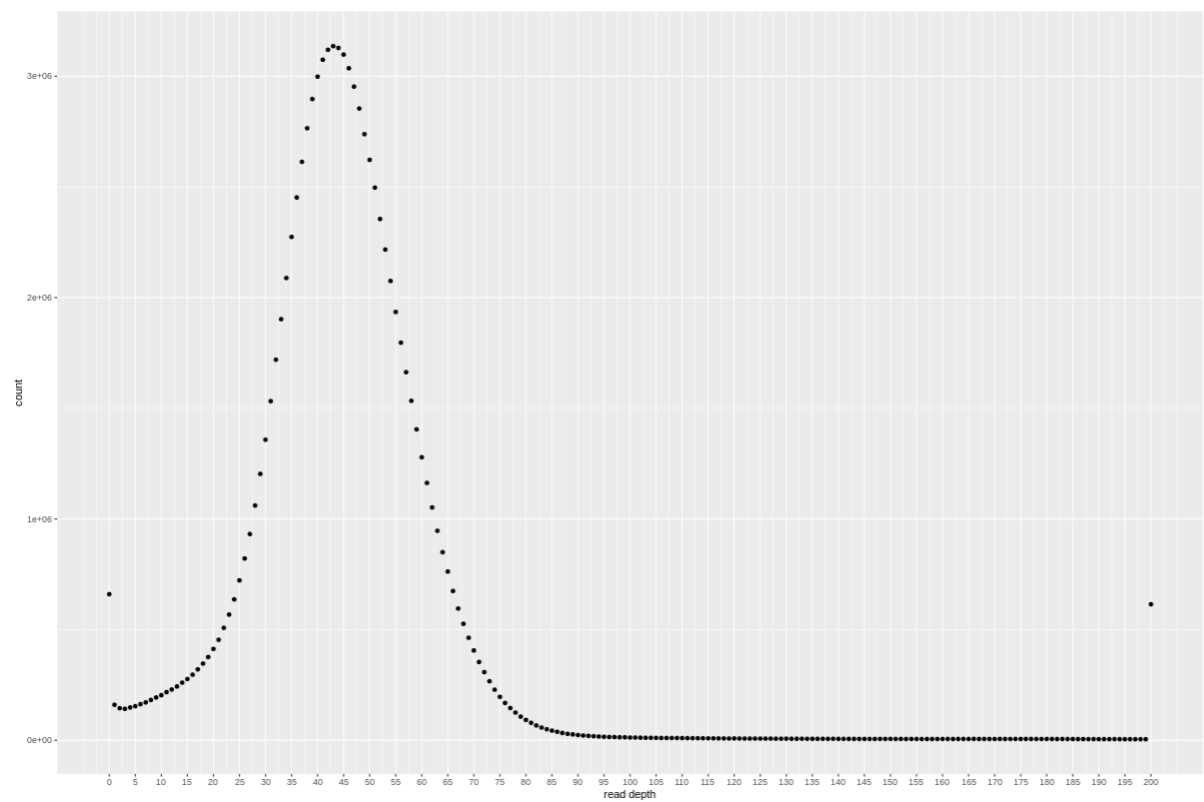**d**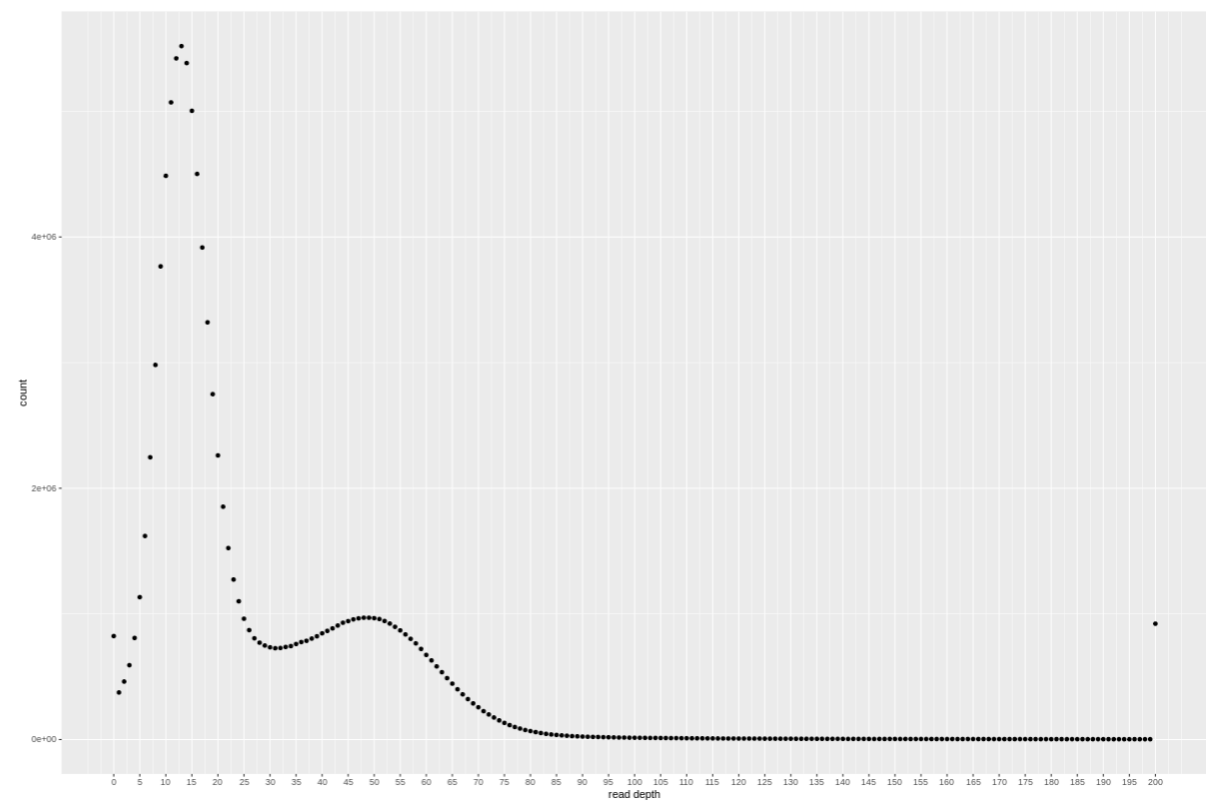

Supplement: jkae181_Supplementary_Data [file jkae181_supplementary_data.zip › Figure_S5_G3-2024-405201.pdf]

**a** Scaffold 1 → 38

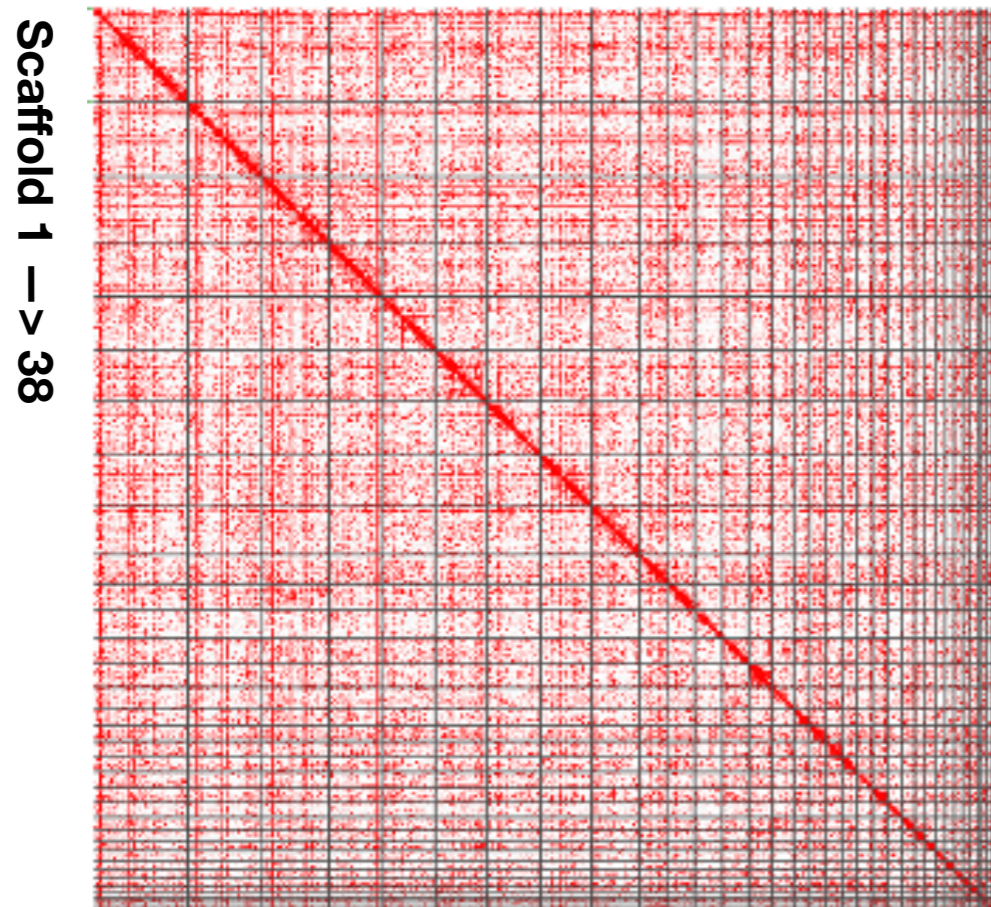

**b** Scaffold 1 → 50

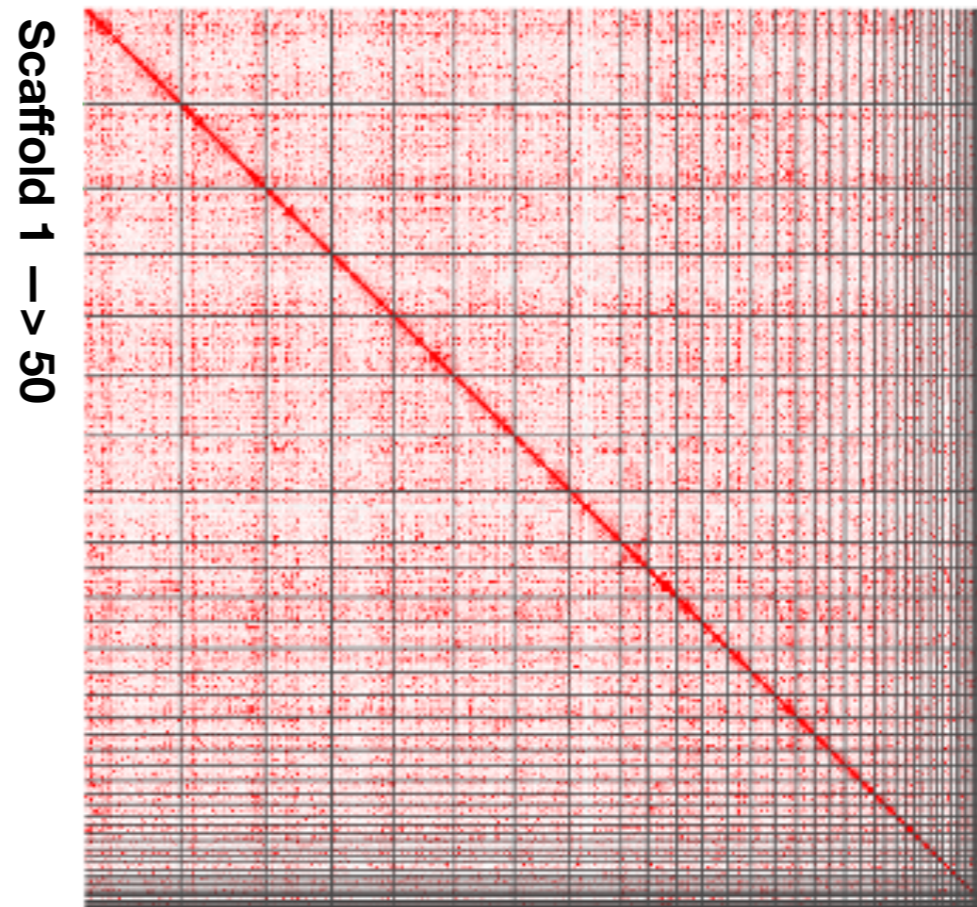

Supplement: jkae181_Supplementary_Data [file jkae181_supplementary_data.zip › Figure_S6_G3-2024-405201.pdf]

**a**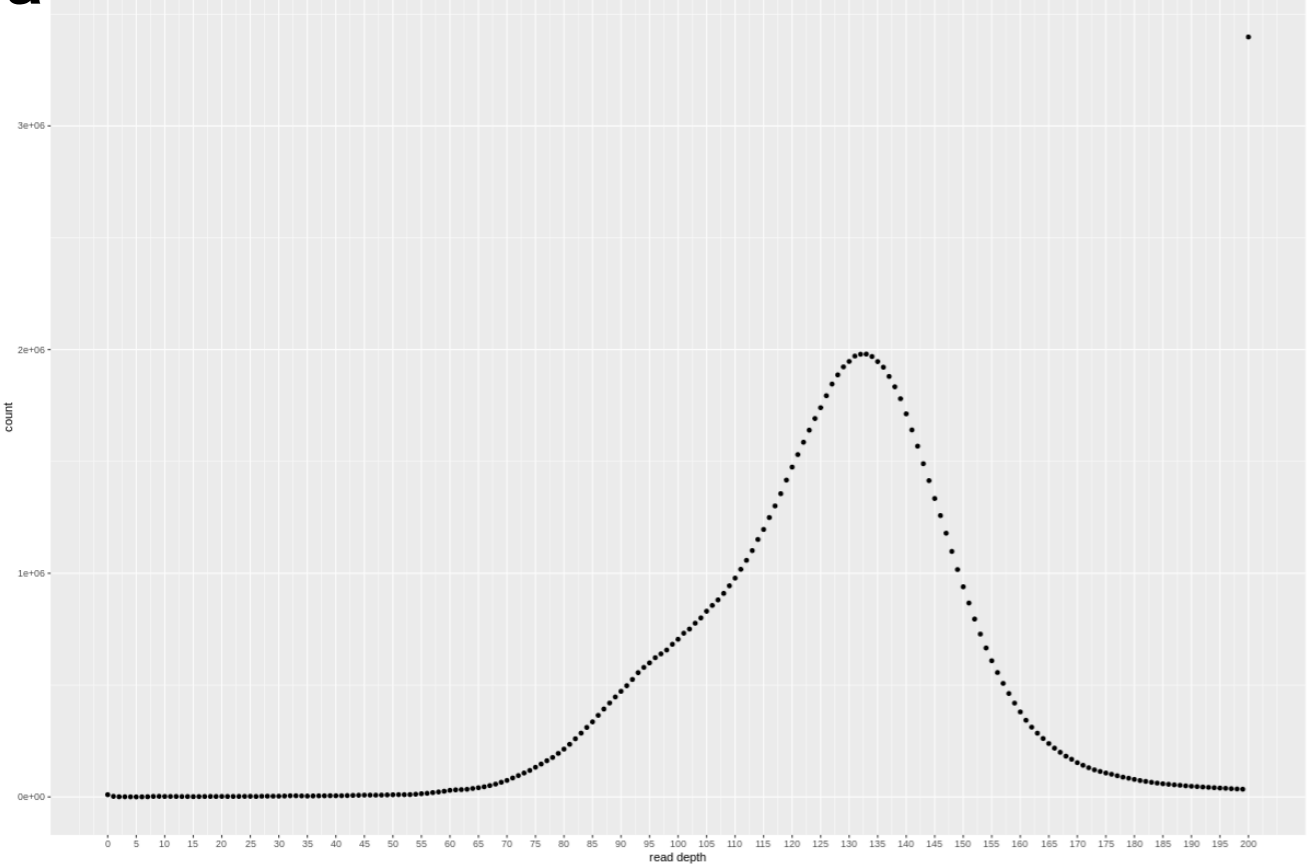**b**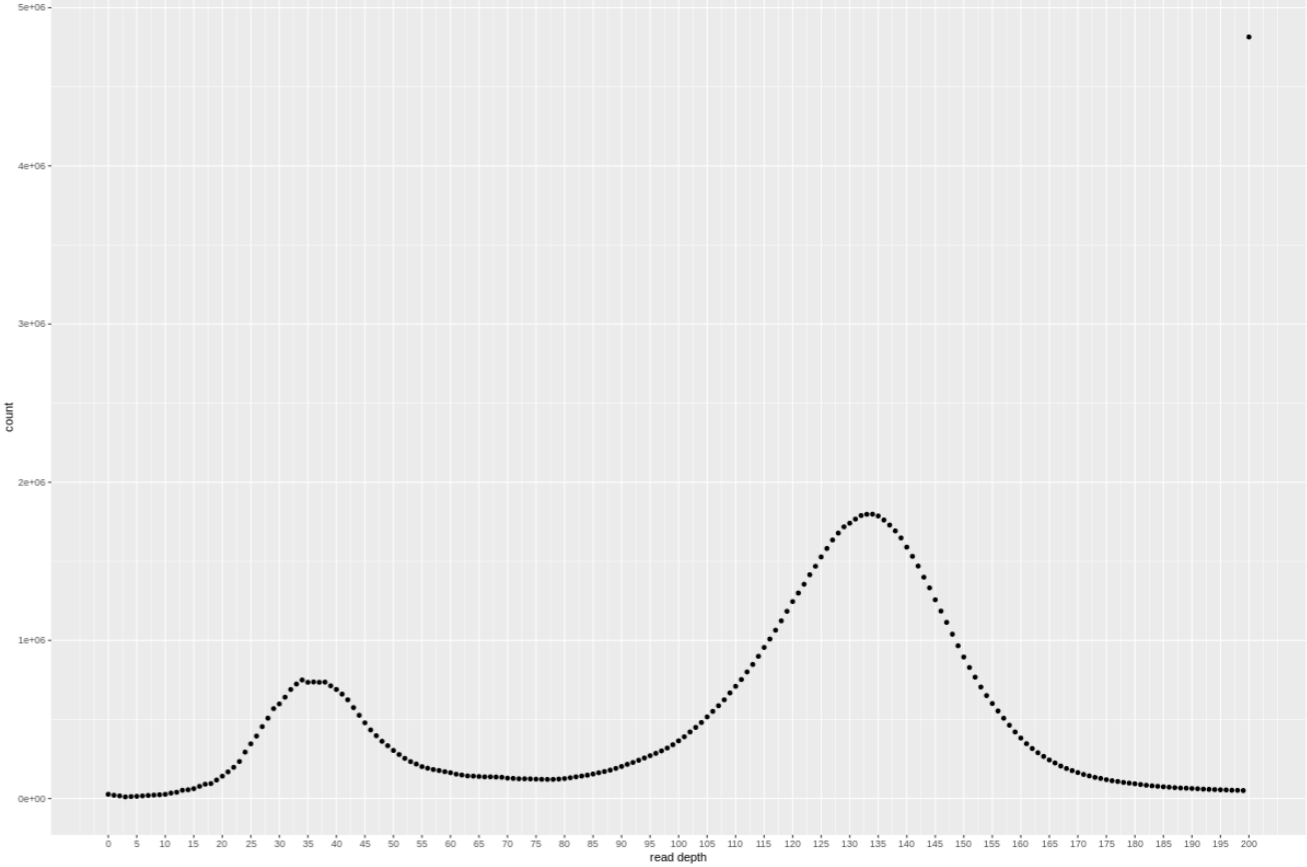**c**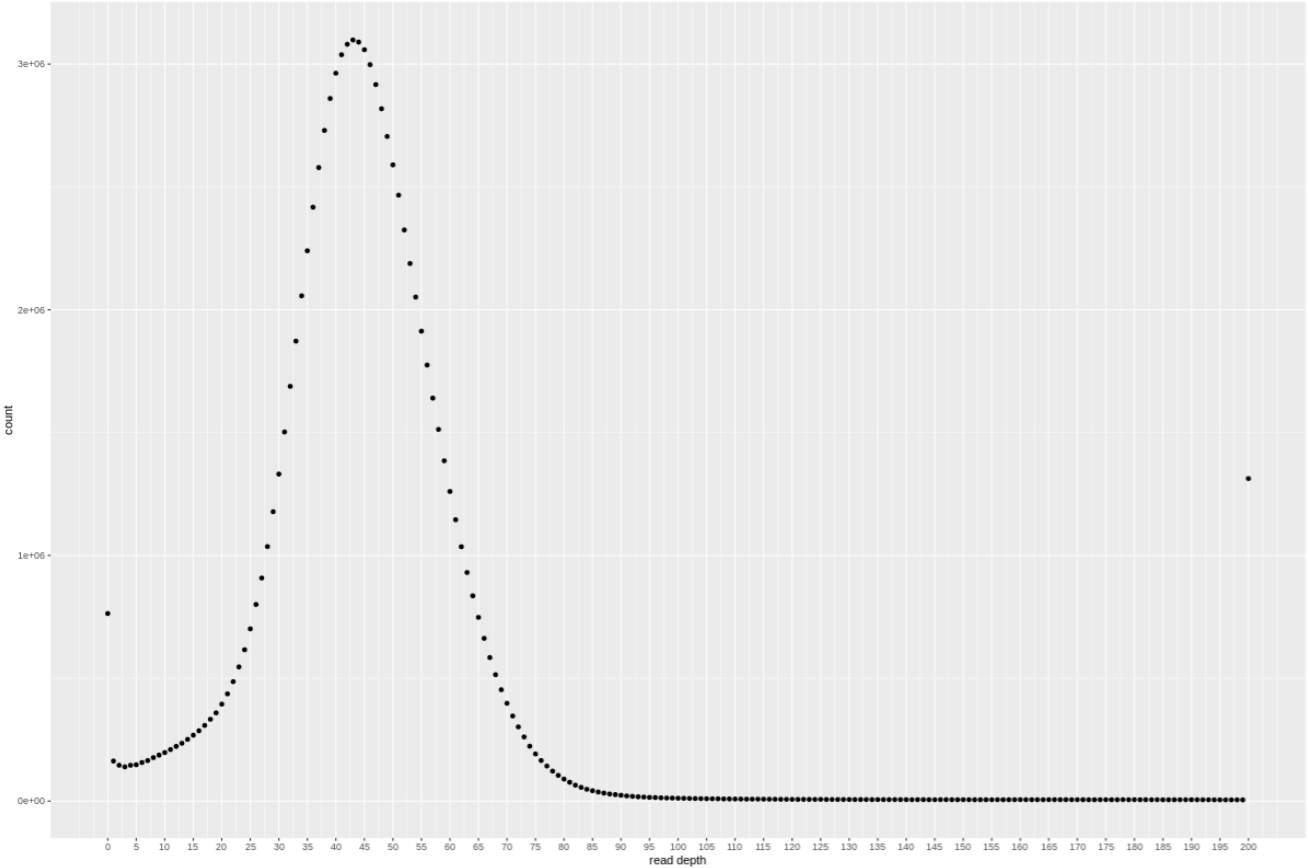**d**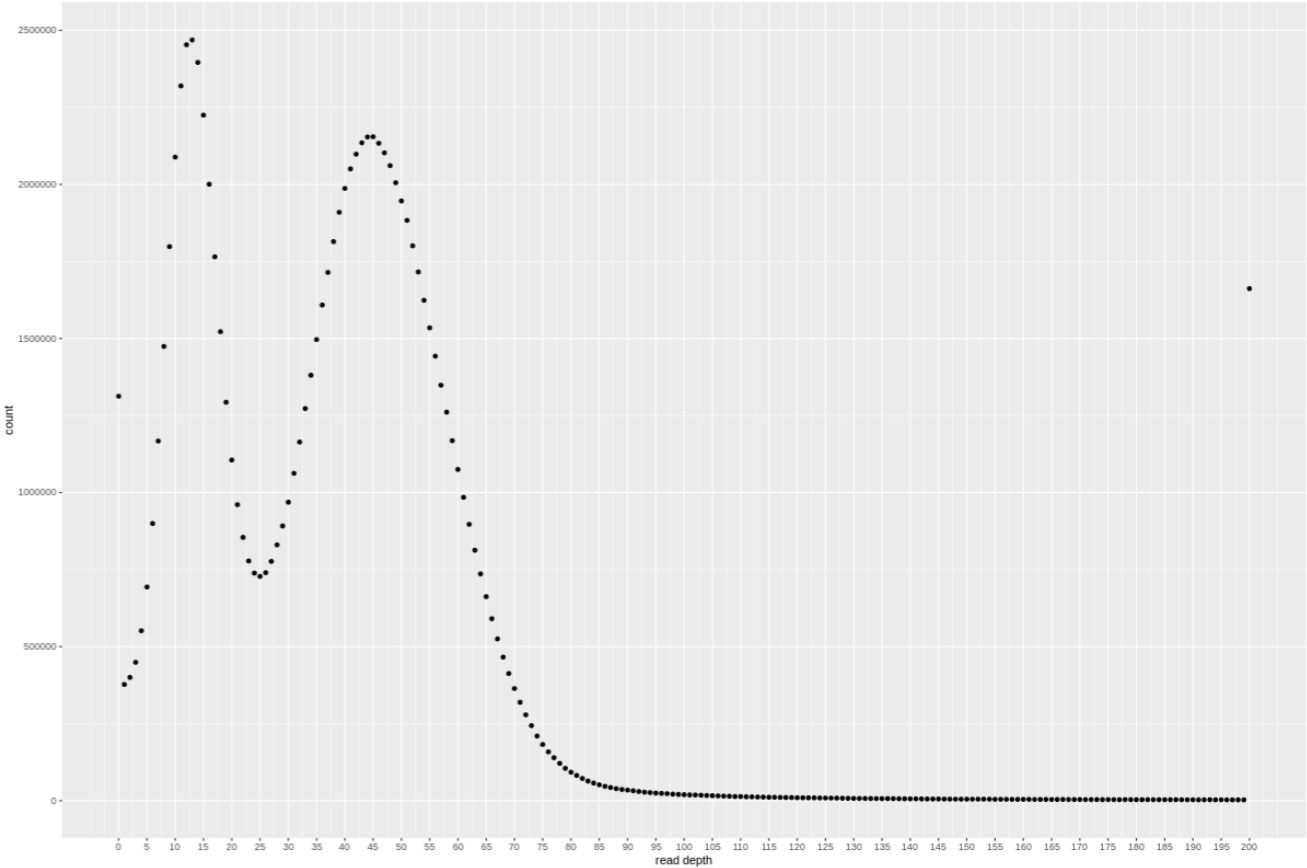

Supplement: jkae181_Supplementary_Data [file jkae181_supplementary_data.zip › Figure_S7_G3-2024-405201.pdf]
